# Histone deacetylase-mediated tumor microenvironment characteristics and synergistic immunotherapy in gastric cancer

**DOI:** 10.7150/thno.86928

**Published:** 2023-08-18

**Authors:** Yilin Lin, Xiangxiang Jing, Zhihua Chen, Xiaoxian Pan, Duo Xu, Xiang Yu, Fengyun Zhong, Long Zhao, Changjiang Yang, Bo Wang, Shan Wang, Yingjiang Ye, Zhanlong Shen

**Affiliations:** 1 Department of Gastroenterological Surgery, Peking University People's Hospital, Beijing 100044, PR China.; 2 Laboratory of Surgical Oncology, Beijing Key Laboratory of Colorectal Cancer Diagnosis and Treatment Research, Peking University People's Hospital, Beijing 100044, PR China.; 3 Department of Gastrointestinal surgery, The First Affiliated Hospital of Fujian Medical University, Fuzhou, Fujian, 350000, PR China.; 4 Department of Radiotherapy, The First Affiliated Hospital of Fujian Medical University, Fuzhou, Fujian, 350000, PR China.

**Keywords:** Histone deacetylases, Gastric cancer, Tumor microenvironment, Immunotherapy, Prognosis.

## Abstract

**Background:** Studies have shown that the expression of histone deacetylases (HDACs) is significantly related to the tumor microenvironment (TME) in gastric cancer. However, the expression of a single molecule or several molecules does not accurately reflect the TME characteristics or guide immunotherapy in gastric cancer.

**Methods:** We constructed an HDAC score (HDS) based on the expression level of HDACs. The single-cell transcriptome was used to analyze the underlying factors contributing to differences in immune infiltration between patients with a high and low HDS. *In vitro* and *in vivo* experiments validated the strategy of transforming cold tumors into hot tumors to guide immunotherapy.

**Results:** According to the expression characteristics of HDACs, we constructed an HDS model to characterize the TME. We found that patients with a high HDS had stronger immunogenicity and could benefit more from immunotherapy than those with a low score. The AUC value of the HDS combined with the combined positive score (CPS)for predicting the efficacy of immunotherapy was as high as 0.96. By single-cell and paired bulk transcriptome sequencing analysis, we found that the infiltration levels of CD4^+^ T cells, CD8^+^ T cells and NK cells were significantly decreased in the low HDS group, which may be induced by MYH11^+^ fibroblasts, CD234^+^ endothelial cells and CCL17^+^ pDCs via the MIF signaling pathway. Inhibition of the MIF signaling pathway was confirmed to potentially enhance immune infiltration. In addition, our analysis revealed that GPX4 inhibitors might be effective for patients with a low HDS. GPX4 knockout significantly inhibited PD-L1 expression and promoted the infiltration and activation of CD8^+^ T cells.

**Conclusion:** We constructed an HDS model based on the HDAC expression characteristics of gastric cancer. This model was used to evaluate TME characteristics and predict immunotherapy efficacy. Inhibition of the MIF signaling pathway in the TME and GPX4 expression in tumor cells may be an important strategy for cold tumor synergistic immunotherapy for gastric cancer.

## Introduction

According to global cancer statistics from 2020, the incidence and mortality rates of gastric cancer rank 5th and 4th among malignant tumors 1, respectively, resulting in a huge economic burden on society 2. The current treatment of gastric cancer involves surgery combined with radiotherapy, chemotherapy, or targeted drug therapy 3. Although patient prognosis has improved, the outcome is still unsatisfactory. Therefore, in-depth exploration of factors affecting the treatment response and prognosis of gastric cancer and formulation of new clinical treatment strategies are the primary tasks for improving patient prognosis.

In recent years, researchers have gained a deeper understanding of the relationship between tumors and the tumor microenvironment (TME). Fleitas *et al*. found that tumor-associated macrophages have great potential as therapeutic targets for gastric cancer 4. Related studies have shown that the heterogeneity of the gastric cancer microenvironment can effectively predict sensitivity to gastric cancer chemotherapy 5. According to previous studies, tumor cells may induce immune escape through the PD-1/PD-L1 signaling pathway 6. In addition, tumor-associated fibroblasts form a high-density extracellular matrix in the TME that blocks drug absorption and immune cell infiltration, leading to different therapeutic responses 7. Zeng *et al*. found that gastric cancer prognosis can be determined by the level of TME cell enrichment 8. Additionally, one study found that clinical MSI-H patients may have a higher immune status and may be more suitable for immunotherapy 9. However, this is not absolute; for example, EBV-positive patients tend to exhibit MSS but have high immune infiltration and can benefit from immunotherapy 10, 11. Therefore, there is no accurate indicator for identifying gastric cancer patients for whom immunotherapy might be beneficial. Therefore, analyzing the heterogeneity of the TME by determining a quantitative index for the TME may be the key to improving the therapeutic effect and prognosis and could guide gastric cancer treatment and prognosis evaluation.

There are reportedly 18 kinds of histone deacetylases (HDAC1-11, SIRT1-7) that play an important role in chromosomal structural modification and gene expression regulation 12, 13. Histone acetylation dissociates DNA and histone octamers, allowing transcription factors and cooperative transcription factors to bind specifically to DNA-binding sites and activate gene transcription. Conversely, deacetylation of histones has the opposite effect 14. HDACs reversibly regulate the acetylation status of histones and nonhistone proteins during TME development 15-17. Moreover, HDAC6 is involved in the upregulation of several key factors in the immune system, such as PD-1 and PD-L1 receptors, which are the main cancer immunotherapy targets 18. Brune *et al*. found that HDAC inhibitor treatment could enhance Foxp3 expression, thereby inducing and maintaining the immunosuppressive Treg phenotype 19. Kai *et al*. found that HDAC3 inhibitors could enhance the differentiation of CD8^+^ T cells into cytotoxic effector cells 20. The above studies indicate that HDACs may be important targets for regulating the TME. However, different types of HDACs present heterogeneity in regulating the infiltration level of TME cells, and a single molecule or a single class of molecular target inhibitors alone cannot precisely control the changes in the TME. Therefore, there is an urgent need to systematically analyze HDAC expression profiles and the corresponding TME characteristics to provide a theoretical foundation for clinical treatment strategies and prognosis evaluation of gastric cancer.

In this study, by analyzing the expression characteristics of HDACs in gastric cancer, an HDAC score (HDS) was established and used to evaluate the microenvironment. We investigated the genomic characteristics of gastric cancer in high and low HDS groups using the multiomics approach, and we found that the high HDS group had a higher degree of immune infiltration and better prognosis. The results were further verified in the bulk transcriptome sequencing cohort of gastric cancer patients at Peking University People's Hospital. This analysis revealed that patients with a high HDS could benefit more from immune checkpoint inhibitor therapy. To explore the internal mechanism affecting the difference in the TME between patients with high and low HDSs, single-cell and paired bulk transcriptome sequencing analysis showed that MYH11^+^ fibroblasts, CD234^+^ endothelial cells and CCL17^+^ pDCs in low HDS samples may inhibit the infiltration of T cells and NK cells through the MIF signaling pathway. In addition, we found that GPX4 inhibitors may be more effective in patients with a low HDS through drug sensitivity prediction. *In vivo* and *in vitro* experiments showed that inhibiting GPX4 expression in tumor cells can significantly enhance the infiltration and activation level of CD8^+^ T cells and improve the prognosis of gastric cancer. In conclusion, we developed an HDS to quantify the TME of gastric cancer to guide the treatment of stratified gastric cancer patients. For patients with a low HDS, this study explored therapeutic strategies to improve the state of the microenvironment and improve prognosis from the aspects of the TME and tumor cells. We believe these findings will contribute to the discovery of new therapeutic targets for gastric cancer and the development of effective therapeutic strategies.

## Methods

### Data acquisition and processing

The RNA-seq, mutation, and somatic copy number variation (SCNV) data, methylation dataset, and clinical information of gastric cancer patients were obtained from the Gene Expression Omnibus (GEO) and TCGA databases. Six cohorts with overall survival information (TCGA-STAD, GSE15459, GSE34942, GSE57303, ACRG, and GSE84437) were included in the study. The RNA-seq data of gastric cancer cell lines were obtained from the Cancer Cell Line Encyclopedia database. The data for pembrolizumab (anti-PD-1) treatment cohorts of gastric cancer patients were obtained from the European Nucleotide Archives (ENA) database (PRJEB25780 and PRJEB40416). Dataset information is shown in Table S1. For microarray data from GEO and other platforms, transcripts per million (TPM) values were used for subsequent analyses. RNA-seq data from the TCGA database were downloaded as fragments per kilobase of exon model per million mapped fragments (FPKM) values, and then the FPKM values were converted into transcripts with transcripts per kilobase of exon model per million mapped reads (TPM). The combat package was used to perform batch correction of data. The mutation data were analyzed using VarScan2 software. Single-cell and paired bulk transcriptome sequencing were provided by Professor Boxi Kang 21.

### Nonnegative matrix factorization (NMF) algorithm for cluster analysis of 18 HDACs

In this study, the expression profiles of 18 HDACs were extracted from six gastric cancer cohorts with prognosis data; these profiles were used to identify the molecular characteristics of gastric cancer mediated by HDACs. The 18 HDACs included four class I HDACs (HDAC1, HDAC2, HDAC3, and HDAC8), six class II HDACs (HDAC4, HDAC5, HDAC6, HDAC7, HDAC9, and HDAC10), seven class III HDACs (SIRT1, SIRT2, SIRT3, SIRT4, SIRT5, SIRT6, and SIRT7), and one class IV HDAC (HDAC11). Based on these findings, NMF algorithm analysis was used to identify different gastric cancer subtypes, and 300 repetitions were performed to ensure the stability of the results 22. The "CancerSubtypes" package was used for the above analysis 23.

### Gene set acquisition and functional annotation

Antigen presentation signatures, immune-related signatures, carcinogenic pathway activation signatures, DNA mismatch repair signatures, and 23 immune cell signatures were obtained from previous studies 24-27. ssGSEA was used to evaluate the score of signatures via the "GSVA" package. The gene set file "c2.cp.kegg.v7.4" from the Molecular Signatures Database was used to determine pathway enrichment scores 28. The R package "ClusterProfiler" was used for the functional annotation of genes 29. The deconvolution algorithm of CIBERSORT was used to analyze the enrichment levels of 22 microenvironmental cells 30.

### Calculation of the HDS in gastric cancer

In the ACRG cohort, the NMF algorithm identified different HDAC clusters. The limma package was used to identify genes that were differentially expressed between different HDAC clusters in the ACRG cohort. An adjusted *P* value < 0.01 was considered to indicate significant differential expression. Univariate Cox regression analysis identified DEGs with significant prognostic value. Only the genes with *P* values < 0.05 were retained. Finally, the Boruta algorithm was used to reduce the dimensionality of DEGs with significant prognostic value 31. Genes with an HR < 1 in univariate Cox regression analysis were defined as gene set A, and genes with an HR > 1 were defined as gene set B. The A and B gene sets were used for subsequent analysis.

PCA was used to construct the HDS, in which principal component 1 was selected as the feature score. Then, we used a method such as GGI to define:



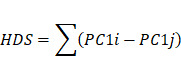



*PC*1*i* represents the feature score of the first principal component of gene A, and* PC*1*j* represents the feature score of the first principal component of gene B. Median HDS scores were used to divide gastric cancer samples into high-HDS and low-HDS groups.

### Whole transcriptome sequencing

A total of 121 gastric cancer tissue samples were ground into powder in liquid nitrogen. The research protocol was approved by the Ethics Committee of Peking University People's Hospital. We defined it as the PKUPH cohort. RNA was extracted from tissues and cells using TRIzol. For the qualified total RNA samples, 1-3 µg of total RNA was used as the starting material for each sample to construct a transcriptome sequencing library. Sequencing was performed using the BGISEQ-500 platform by running a paired-end sequencing program (PE), and 150 bp paired-end sequencing reads were obtained. TPM values were used to calculate gene expression levels. Statistical methods are described in previous studies 32.

### Multiple immunofluorescence staining assay

Forty-seven cancer tissue specimens with transcriptome sequencing data were included in this study. The patients did not undergo neoadjuvant treatment prior to surgery. The research protocol was approved by the Ethics Committee of Peking University People's Hospital. The tissue block sections were cut into 3 µm lengths and embedded in paraffin before being placed on a glass slide. The paraffin sections were dewaxed, treated with EDTA antigenic repair buffer in a microwave oven for antigenic repair and then sealed with serum. The sealing solution was gently shaken off, and PBS was added to the section in a certain proportion of the first primary antibody and incubated at 4 °C overnight. The corresponding HRP-labeled secondary antibody was added for incubation at room temperature, and the fluorescence enhancer was added dropwise. After incubation at room temperature and away from light, microwave treatment was carried out. The second primary antibody and the third primary antibody were added in accordance with the above steps. After DAPI staining of the nucleus, the tablets were sealed with anti-fluorescence quenching tablets. Antibodies against CD8α (ab237709), CD4 (ab183685), pan-CK (ab7753) and PD-L1 (ab213480) were purchased from Abcam. The slices were placed under a scanner to capture images. The proportion of positive cells was calculated.

### Single-cell transcriptome sequencing analysis

A Seurat matrix was constructed after cells were identified with CellRanger, low-quality cells were filtered according to previous studies 21, and finally, data were obtained for cluster analysis. First, the top 2000 genes with the highest variance were selected for data normalization, principal component analysis (PCA) was used to reduce the dimensionality of the data to 50 principal components, and the harmony function was used to remove the batch effect of the samples. A total of 9 clusters (B cells, CD4^+^ T cells, CD8^+^ T cells, NK cells, mast cells, endothelial cells, fibroblasts, myeloid cells, and plasma cells) were identified by tSNE cluster analysis. Then, the gene expression matrix of each cell group was extracted for the identification of subpopulations. The top 2000 genes in terms of variance were used for principal component analysis, and the top 25-30 principal components were used for batch correction with Harmony. The Wilcoxon rank sum test was used to identify differentially expressed genes between subpopulations. Finally, ligand and receptor information in the CellChat library was used to analyze the communication of each subgroup of cells 33.

### Prediction of gastric cancer immunotherapy response and chemotherapeutic drug sensitivity

The ISOpureR package was used to purify nontumor tissue data from our bulk sequencing data 34. The CTRP2.0 and PRISM databases were used to predict sensitivity to chemotherapeutics 35, 36. These two databases defined the sensitivity of tumors to drugs by evaluating their AUC values. Based on PRISM and CTRP2.0 drug sensitivity AUC data and CCLE expression profile data, potential therapeutic drugs were predicted for high and low HDS tumor cell characteristics.

### Cell culture and transfection

The AGS and MFC cells were cultured in RPMI 1640 medium (Gibco, USA) containing 10% fetal bovine serum (FBS) (Gibco, USA) at 37 °C and 5% CO_2_. Three si-GPX4s were purchased from Gemma (Shanghai, China). AGS cells were seeded in 6-well plates, and siRNA transfection experiments were performed when the cell density reached 80%. Transfection was performed according to the manufacturer's instructions. RNA was extracted 48 hours after transfection for subsequent analysis. The siRNA sequence was shown in Table S6.

### Construction of lentiviral knockdown GPX4 cell lines

The mouse GPX4 knockdown lentivirus was purchased from GeneChem (Shanghai, China). MFC cells were seeded in 48-well plates, and a lentivirus transfection assay was performed according to the reagent manufacturer's instructions.

### Western blot assay

Cells were collected and fully lysed with RIPA lysis buffer containing 1% phenylmethanesulfonyl fluoride. Protein loading buffer was then added to the protein sample and boiled in a 100 °C water bath for 5 min. The protein was then subjected to SDS‒PAGE and transferred to a polyvinylidene fluoride (PVDF) membrane after electrophoresis. Then, PVDF was incubated in 5% skimmed milk powder for 2 h, and the primary antibody (GPX4, GAPDH or PD-L1) was incubated at 4 °C overnight after washing. After the PVDF membrane was washed three times again, the secondary antibody with HRP was incubated for 2 h. Finally, enhanced chemiluminescence (ECL) reagent was added to the PVDF membrane, and the bands were exposed on a Bio-Rad instrument.

### Real-time quantitative polymerase chain reaction (RT‒qPCR) assay

RNA was extracted using TRIzol. The reverse transcription and amplification kits were purchased from TransGen Biotech (China). The methods were performed according to the reagent manufacturer's instructions. GAPDH was used as an internal reference gene. The 2^-∆∆CT^ method was used to analyze the relative expression of genes.

### CD8^+^ T-cell stimulation assay

The 6-well plates were coated with 3 μg/mL anti-CD3 (BioLegend, cat 100359) and incubated at 37 °C for 2 h. CD8^+^ T cells were isolated from the spleens of 615 mice using the STEMCELL EasySep mouse CD8^+^ T-Cell Isolation Kit. The fluid in 6-well plates was removed, and 1×10^6^ CD8^+^ T cells were cultured in each well and then in complete medium 1640 containing 2 μg/mL anti-CD28 antibody (BioLegend, cat 102116) for 48 h.

### T-cell coculture assay

A total of 1×10^5^ cells per well were inoculated into 96-well plates and incubated for 2 h. The activated CD8^+^ T cells and tumor cells were cocultured in 1640 medium containing 100 U/mL IL-2 (PEPROTECH), 10% FBS, 10 mM HEPES, 100 μM NEAA and 50 μM β-mercaptoethanol at a ratio of 5:1 for 48 h. Two hours before cell collection, Brefeldin A (BioLegend, 1:1000) was added to block cytokine secretion. The cells were washed and resuspended in a dye buffer, stained with anti-CD8 α and incubated in the dark on ice for 30 min. After washing, the cells were fixed, broken and stained on ice for 30 min with anti-IFN-γ or anti-GZMB antibodies. The cells were suspended and analyzed by flow cytometry.

### Cell proliferation, apoptosis, migration, and invasion assays

The cells were seeded in six-well plates and stained with the EdU kit (Beyotime, C0075S) and apoptosis kit (Multi Science, AP107-100). The stained cells were suspended and detected by flow cytometry. The cells were resuspended in 1640 medium without FBS. A total of 2×10^5^ or 5×10^5^ cells were seeded in the upper layer of transwell chambers without or with Matrigel. The lower chamber layer was supplemented with 1640 medium containing 10% FBS. After coculture for 48 or 72 h, the cells were fixed with methanol. Cells in the upper chamber were removed, and the remaining cells were stained with 2% crystal violet. Finally, images were taken under the microscope, and the migrating and invading cells were counted.

### Mouse model of subcutaneous tumors

When the cell confluence reached 80%, 1×10^6^ sh-NC/sh-GPX4 MFC cells were resuspended in PBS and injected subcutaneously into the right oblique abdomen of 615 mice (Female, 6-8 weeks old, Wukong biology). Anti-mouse-PD-L1 (BioXcell) or MIF inhibitor (4-IPP, 80 mg/kg, MCE) was used for intraperitoneal therapy. Tumor growth and mouse survival were monitored by *in vivo* imaging, and the tumor volume was calculated as a×b^2^/2 (a is the longest diameter of the tumor, b is the shortest diameter of the tumor). Mice with tumors smaller than 1000 mm^3^ were considered viable. Tumor tissues were collected and cut into small pieces (1~2 mm^3^) with eye scissors. Protease solution (1 mg/mL collagenase D and 0.1 mg/mL DNase I, Thermo Fisher Scientific) was added for digestion in a 37 ℃ incubator for 30 min. After termination of digestion, the cells were filtered through a 70 μm cell filter. The cells were washed once with dye buffer. Anti-CD45, anti-CD8α or anti-CD4 antibodies (Biolegend) were used to stain the cells, and the proportions of CD8^+^ T cells and CD4^+^ T cells were detected by flow cytometry. Intracellular staining was performed as described above. All animal experimental protocols of this study were approved by the Ethics Committee of Peking University People's Hospital.

### Statistical analysis

All statistical *P* values were two-sided, and *P* < 0.05 was considered to indicate statistical significance. All analyses were performed using R software (version 4.0.2).

## Results

### Landscape of HDACs in gastric cancer

**Figure 1:**
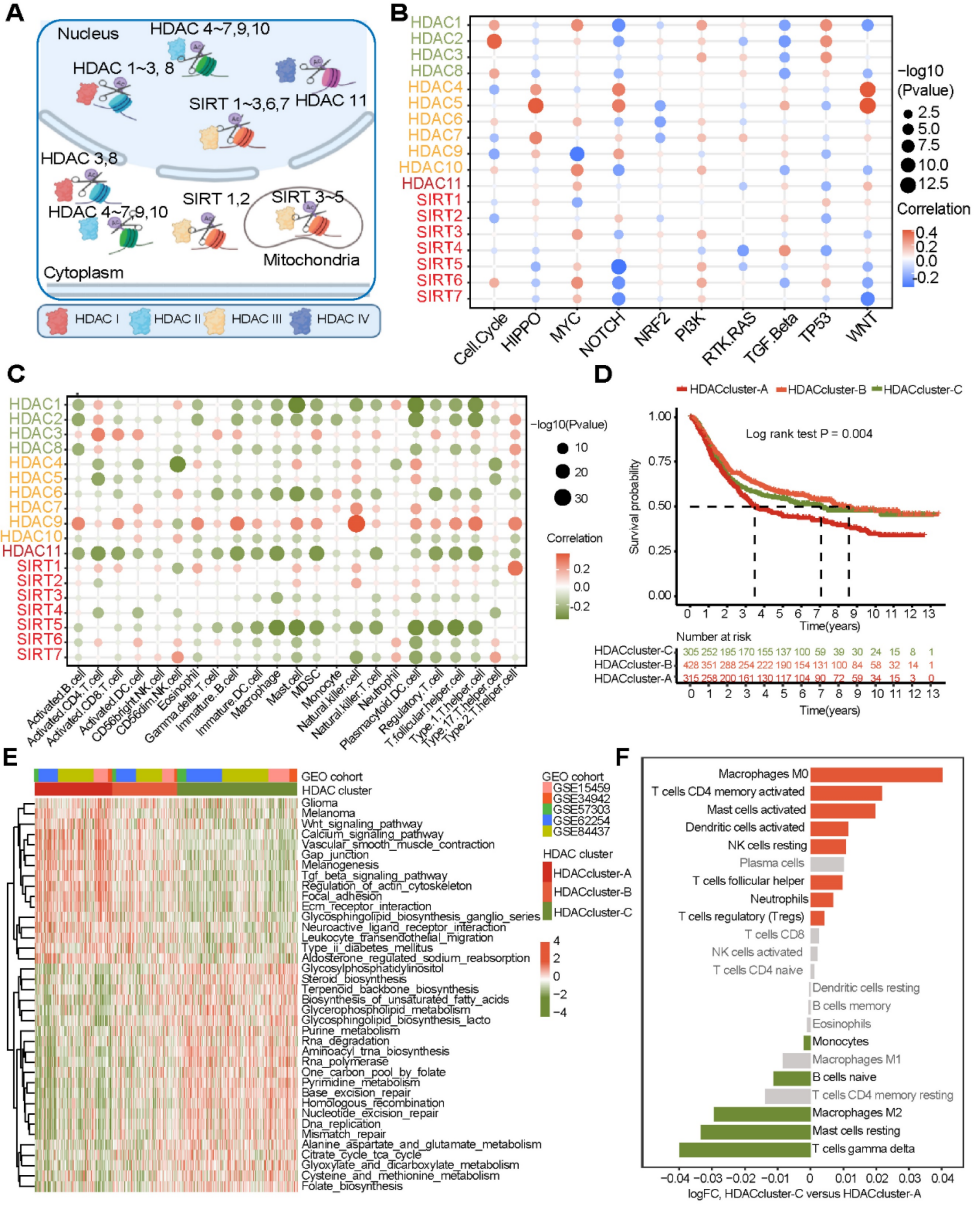
** Landscape of histone deacetylases (HDACs) in gastric cancer (GEO cohort). (A)** The mechanism of HDACs in cells. **(B)** Association of HDAC expression with ten cancer-related pathways. **(C)** Correlation between HDAC mRNA expression levels and immune cell infiltration. **(D)** Three gastric cancer subtypes with significant prognostic differences as identified by the mRNA expression of HDACs. (E) KEGG functional enrichment analysis to understand the characteristics of different HDAC clusters. **(F)** The difference in TME cell infiltration levels between different HDAC clusters (CIBERSORT algorithm).

Figure 1A shows the location of the 18 HDACs and mechanisms regulating protein acetylation. In the TCGA database, we found that the most common mutations of class II HDACs co-occurred in gastric cancer (Figure S1A). We then analyzed the relationship between HDAC mutations and mRNA expression. We found that mutations in HDAC4, HDAC5, and HDAC9 significantly decreased the mRNA expression levels of the corresponding HDACs (Figure S1B).

Since copy number variants (CNVs) are also known to alter gene expression, we further analyzed the CNV status of 18 HDACs in gastric cancer and found that CNVs of HDACs were often observed and were different for various HDACs (Figure S1C). In addition, for HDACs showing CNVs, we analyzed the corresponding mRNA expression levels. The results showed that except for HDAC5, HDAC6, HDAC7, and HDAC9, the copy number loss of the other 14 HDACs significantly inhibited the expression of the corresponding mRNA. In contrast, the copy number gain of these 14 HDACs significantly promoted the expression of the corresponding mRNAs (Figure S1C).

We included five GEO datasets with prognostic information (GSE15459, GSE34942, GSE57353, ACRG, and GSE84437) in this study. The combat package was used to perform batch correction of data. A total of 1048 gastric cancer samples with prognostic information were included (Table S1). To explore the role of HDACs in gastric cancer, we enriched the scores of ten cancer-related pathways in the GEO dataset through the ssGSEA method. We found that the expression of HDACs was significantly related to ten cancer signaling pathways. However, the correlation between HDAC expression and cancer-related pathway enrichment was not consistent, even for HDACs in the same class (Figure 1B).

### HDAC-mediated subtype characteristics in gastric cancer

Next, we analyzed some gastric cancer subtypes that have already been identified. We found that the expression of HDAC was heterogeneous among the four subtypes of the TCGA cohort (CIMP-EBV, CIMP-H, CIMP-L, and non-CIMP) (Figure S2A). This result was also observed in other subtypes (CIN, EBV, GS, and MSI) (Figure S2B). Moreover, Aggarwal *et al*. 37 divided gastric cancer into four subtypes (EMT, MSI, MSS/TP53-, and MSS/TP53+) based on Asian populations. Our results also revealed that the expression of HDACs significantly differed based on gastric cancer subtypes (Figure S2C). MSI status and EBV status are important indicators of gastric cancer outcomes. We analyzed the expression of HDACs in samples of different MSI and EBV statuses (from the TCGA and ACRG cohorts). Our results verified that HDACs were significantly differentially expressed in the MSI, non-MSI, EBV+, and EBV- subtypes (Figure S2 E-F). These gastric cancer subtypes have been shown to be related to the TME state. Then, we found that the expression of HDACs was significantly related to the cell infiltration level in the TME, but significant heterogeneity was observed (Figure 1C).

These results reveal that the expression of HDACs may play important but heterogeneous roles in gastric cancer. Therefore, assessment of the expression levels of all HDACs rather than just a few molecules may help to accurately identify the characteristics of gastric cancer and provide new treatment strategies.

### Nonnegative matrix factorization for constructing a new classification system for gastric cancer based on the expression of HDACs

We analyzed the relationship between the expression of the 18 HDACs and the prognosis of gastric cancer in the GEO cohort (GSE15459, GSE34942, GSE57353, GSE62254, and GSE84437). Univariate Cox regression analysis revealed that HDAC1, HDAC5, HDAC8, SIRT3, and SIRT6 were significant predictors of gastric cancer (Figure S3A and Table S2).

**Figure 2:**
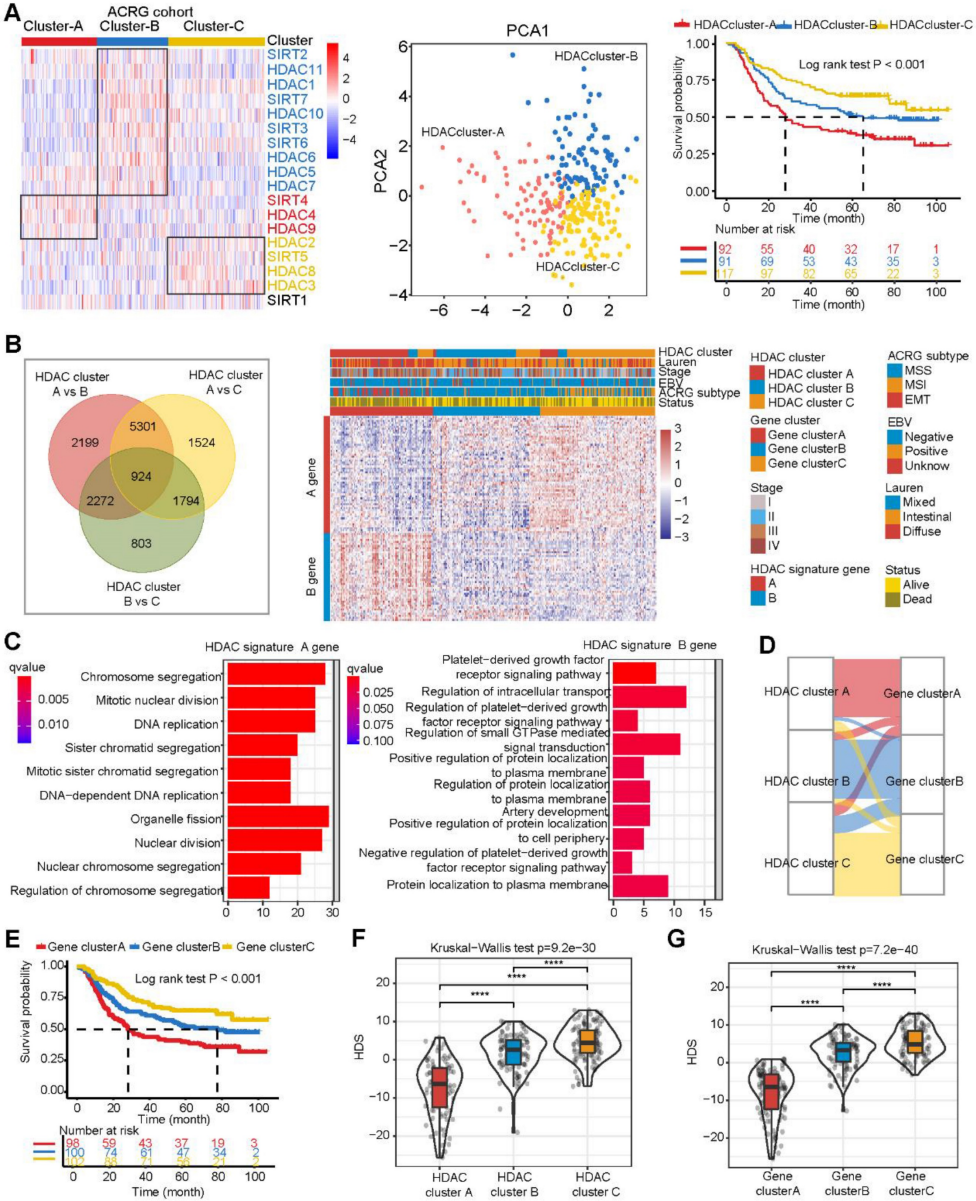
** Construction of the histone deacetylase score (HDS) model in the ACRG cohort. (A)** Differential expression of HDAC mRNAs in the three HDAC clusters. Principal component analysis revealed the distribution of the three HDAC clusters. Prognostic differences between different HDACs. **(B)** Differentially expressed genes in the three HDAC clusters. The heatmap shows the expression profile of 103 genes (A genes and B genes) in the ACRG cohort after dimensionality reduction by the Boruta algorithm. **(C)** The biological processes of HDAC signature A genes and B genes. **(D)** Sankey diagram showing the relationship between three HDAC clusters and three gene clusters. **(E)** Prognostic differences between different gene clusters. **(F)** Construction of the HDS model through principal component analysis and evaluation of the difference in the HDS between different HDAC clusters and gene clusters. *P < 0.05, **P < 0.01, ***P < 0.001, ****P < 0.0001.

Next, NMF clustering was used to classify gastric cancer into 2-5 subtypes based on the expression profile of HDACs, and three clusters were found to be the most appropriate (Figure S3B). The heatmap shows that these three clusters (Figure S3C) can be used to stratify patients based on the expression of HDACs. HDACs were differentially expressed in the three clusters (Figure S3D). The survival curve revealed that the overall survival of gastric cancer patients was significantly different in these three clusters (Figure 1D), and HDAC cluster A patients had the worst prognosis. To further analyze the role of the three clusters in gastric cancer, KEGG enrichment analysis was used to identify the pathways enriched in the three clusters. The limma package was used to identify pathways that were significantly differentially enriched between cluster pairs. The results revealed that multiple oncogenic pathways, including glioma, melanoma, WNT, and TGF-β signaling pathways, were activated in HDAC cluster A (Figure 2E and Table S3). In addition, ECM receptor interactions and focal adhesion pathways were also enriched in HDAC cluster A. The activation of metabolic pathways and DNA damage repair pathways in HDAC cluster C had a high enrichment score, and this score was slightly lower in HDAC cluster B.

Finally, we used the deconvolution algorithm CIBERSORT to estimate the immune cell enrichment level in each sample again and further compared the immune infiltration level between HDAC cluster A and HDAC cluster C. The results revealed that CD4 memory cells, mast cells, and DCs were activated in HDAC cluster C, but T-regulatory cells were also enriched. In HDAC cluster A, M2 macrophages were enriched.

According to the above results, gastric cancer subtypes had different levels of HDAC expression, and cell infiltration in the TME was broadly regulated by HDACs.

### Characteristics of HDAC clusters in the ACRG cohort

The above results were analyzed based on five GEO datasets. Even if the batch effect removal method was applied, the heterogeneity between the datasets may still have affected the display of the results. For this reason, we focused on one of the largest datasets, the ACRG cohort dataset. This dataset contained a large amount of complete clinical information. We performed NMF cluster analysis on the expression profiles of HDACs in the ACRG cohort. The samples could be divided into three stable subtypes (HDAC clusters A, B, and C) (Figure S4A). HDAC expression profiles in these three subtypes were analyzed. HDACs were significantly differentially expressed in these three subtypes (Figure S4B). The heatmap clearly shows that SIRT4, HDAC4, and HDAC9 were highly expressed in cluster A; HDAC1, HDAC5-7, HDAC10-11, SIRT2-3, and SIRT6-7 were highly expressed in cluster B; and HDAC2, HDAC3, HDAC8, and SIRT5 were highly expressed in cluster C (Figure 2A). We performed PCA based on the HDAC clusters and found that these three clusters could distinguish gastric cancer samples effectively (Figure 2A). Survival analysis revealed that the overall survival of the three clusters was significantly different. HDAC cluster A had the worst prognosis in terms of overall survival, while HDAC cluster C had the best prognosis (Figure 2A).

### Calculation of the HDS to quantify the characteristics of patients with gastric cancer

To better understand the transcriptome characteristics of different HDAC clusters, we used the limma package to identify genes that were significantly differentially expressed between different HDAC clusters based on the ACRG cohort, with *P* < 0.01 indicating a significant difference. A total of 924 genes were differentially expressed among the three clusters (Figure 2B). Next, using univariate Cox regression analysis, we identified 488 genes significantly associated with gastric cancer prognosis. Then, the Boruta algorithm was used to perform gene dimensionality reduction, and a total of 103 genes were finalized. Univariate Cox regression results for 59 genes showed that the hazard ratios (HRs) were all < 1 (Table S4). High expression levels of these genes implied a good prognosis. We defined these genes as type A genes. In contrast, the univariate Cox regression results of 44 genes revealed that their HRs were all > 1 (Table S4). High expression levels of this type of gene implied a poor prognosis. We defined these genes as type B genes. We performed NMF cluster analysis on these 103 genes and found that samples of gastric cancer could also be divided into three gene clusters (Figure S5A and Figure 2B). Moreover, HDAC expression and immune cell infiltration levels were significantly different among these three gene clusters (Figure S5B). GO analysis revealed that the HDAC signature A genes were mainly enriched in cell differentiation and cycle regulation, and the HDAC signature B genes were mainly enriched in protein localization and regulation (Figure 2C). The Sankey diagram shows the relationship between the three gene clusters and three HDAC clusters (Figure 2D). This result reveals that gene clusters and HDAC clusters are highly consistent. Moreover, survival curve analysis further demonstrated that the overall survival associated with samples in the three gene clusters was significantly different, wherein gene cluster C indicated the best prognosis and gene cluster A indicated the worst (Figure 2E). These results suggest that the 103 genes could effectively represent the characteristics of HDAC clusters in gastric cancer.

However, it is difficult to apply these classifications in clinical practice. Therefore, based on the expression of 103 genes, we constructed an HDS model and calculated the score of each patient. The process for this method is shown in Figure S5C. The HDS value for each sample is shown in Table S5. The Kruskal‒Wallis test results revealed that the HDS had significant differences in certain HDAC clusters and gene clusters (Figure 2F-G). This result was consistent with the HDAC clusters and gene clusters.

### Clinical features and genomic characteristics of the HDS in gastric cancer

**Figure 3:**
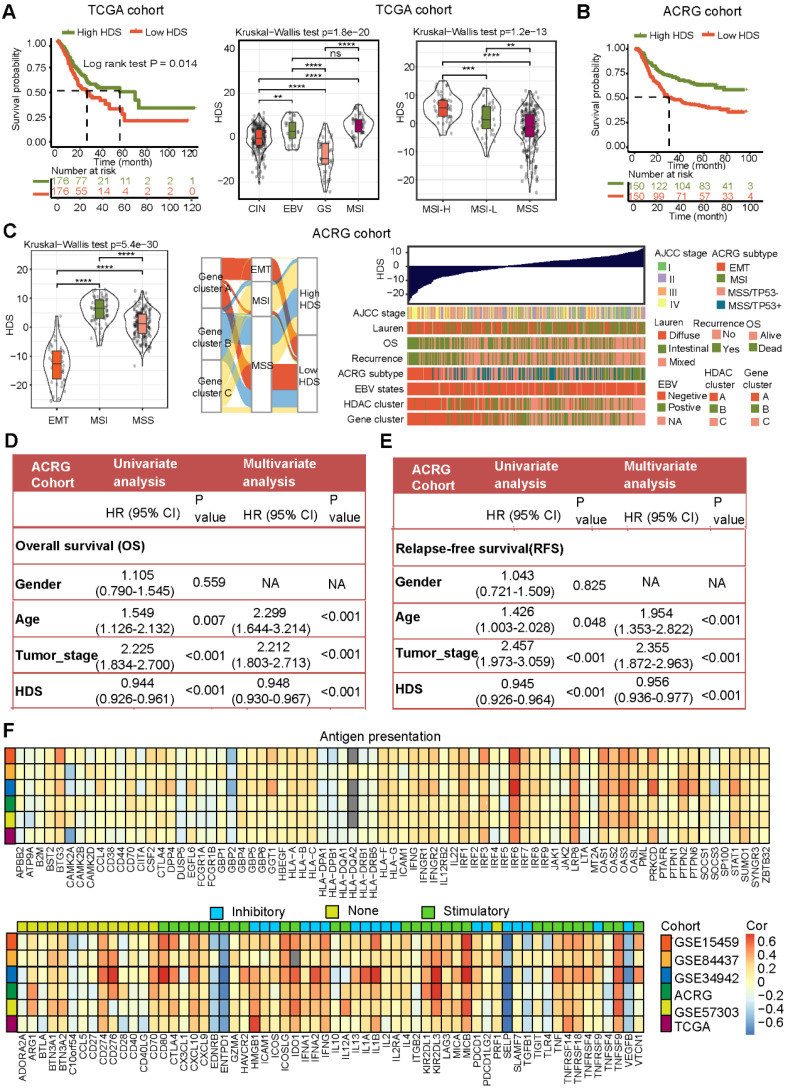
** Clinical features and genomic characteristics associated with the HDS in gastric cancer. (A)** The prognostic difference between the high- and low-HDS groups (TCGA cohort). Differences in the HDS in gastric cancer subtypes (TCGA cohort). **(B)** The prognostic difference between the high- and low-HDS groups (ACRG cohort). **(C)** Differences in the HDS in gastric cancer subtypes and clinical features (ACRG cohort). **(D-E)** Univariate and multivariate COX regression were used to identify the effects of the HDS and clinical features on overall survival (OS) and relapse-free survival. **(F)** The correlation between the HDS and antigen presentation molecules, costimulatory molecules, and coinhibitory molecules. *P < 0.05, **P < 0.01, ***P < 0.001, ****P < 0.0001.

We attempted to determine the relationship between the HDS and prognosis and clinical characteristics. We divided patients with gastric cancer into high and low HDS groups based on the median score. The results revealed that the high HDS group had a longer survival time than the low HDS group in the TCGA cohort (Figure 3A). In addition, we found that the MSI-H- and EBV-positive subtypes had the highest HDS. Similarly, we observed in the ACRG cohort that a low HDS was associated with a worse prognosis than a high HDS (Figure 3B). The MSI subtype had the highest HDS, whereas the EMT subtype had the lowest HDS (Figure 3C). The Sankey diagram shows the relationship between the HDS in gene clusters and ACRG subtype characteristics (Figure 3C). The waterfall chart shows the relationship between the HDS and clinical features in the ACRG cohort (Figure 3C). Then, we analyzed the prognosis of patients with a high and low HDS in different GEO cohorts (Figure S6A). These results demonstrate that patients with a high HDS had a better prognosis and a longer recurrence-free survival time (Figure S6B). Finally, we explored whether the HDS was a prognostic marker for gastric cancer. Cox regression analyses revealed that the HDS was an independent prognostic factor for gastric cancer (Figure 3D).

The above results revealed that HDAC expression, HDAC clusters, and gene clusters were significantly related to the TME and that the HDS was also related to immune-related subtypes (such as EBV subtypes and MSI-H subtypes). These results indicate that the HDS may indirectly reflect the TME characteristics of gastric cancer. Then, we analyzed the correlation between the HDS and the expression of antigen presentation-related molecules, costimulatory molecules, and coinhibitory molecules in six gastric cancer cohorts. We found that the HDS was positively correlated with the expression of most antigen-presenting molecules (Figure 3F). This result suggests that the high HDS group had a significantly higher antigen-presenting ability than the low HDS group. Further analysis revealed a positive correlation between the HDS and the expression of costimulatory molecules and cosuppressive molecules (Figure 3F). This result demonstrates that patients with a high HDS value had higher immunogenicity, but at the same time, there was stronger immunosuppression.

### High HDS may indicate “hot” tumor status in gastric cancer

**Figure 4:**
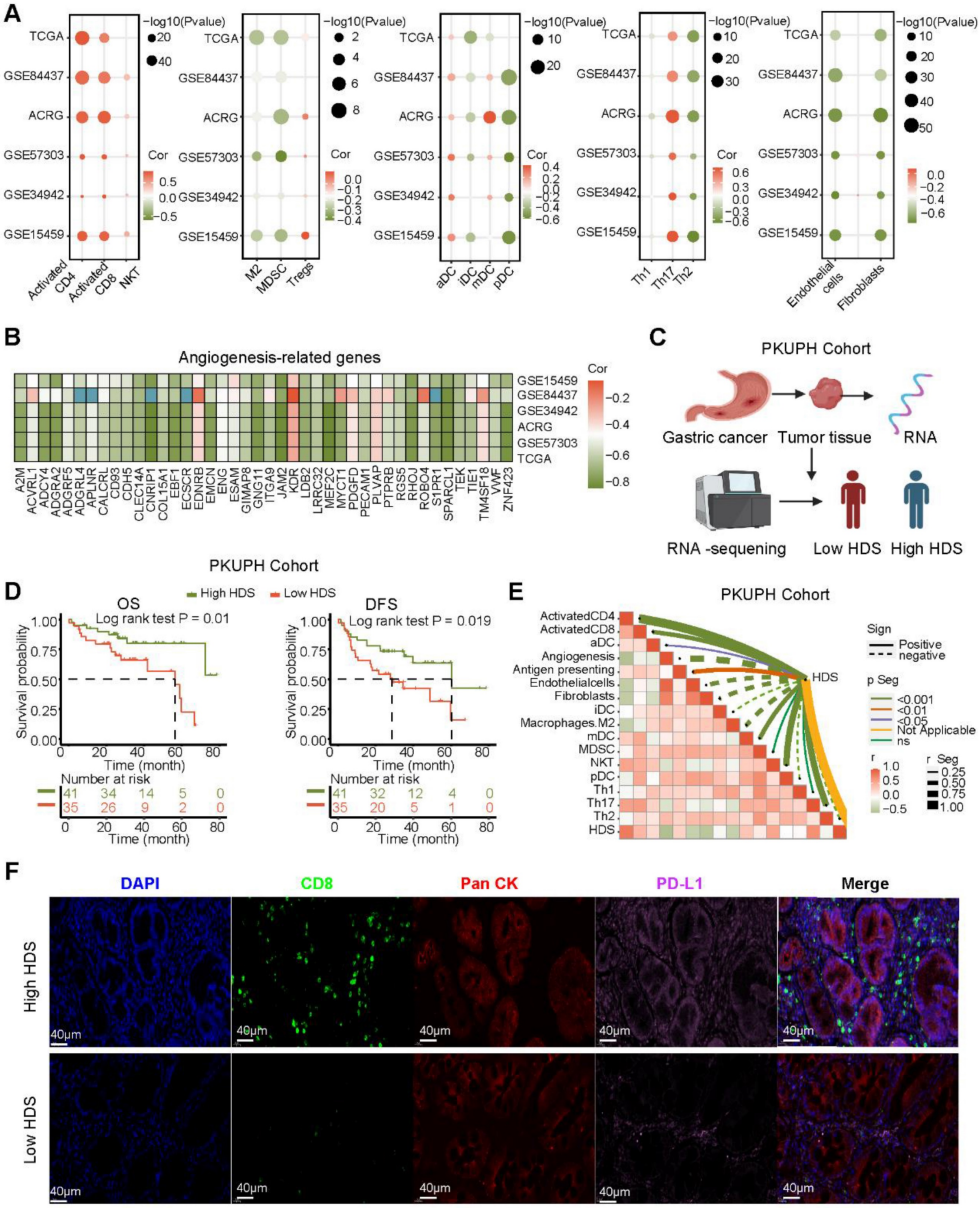
** Association of immune characteristics with the HDS. (A)** The correlation between the HDS and TME cell enrichment scores was analyzed in five gastric cancer cohorts. **(B)** The HDS was significantly negatively correlated with the expression of angiogenic molecules in five gastric cancer cohorts. **(C)** Flow chart of RNA-seq. **(D)** Survival analysis revealed that patients with a high HDS had longer OS and DFS. **(E)** The correlation between the HDS and TME cell enrichment scores was analyzed in the PKUPH cohort. **(F)** The expression levels of CD8 and PD-L1 proteins in high- and low-HDS samples.

To investigate the relationship between the HDS and TME, we analyzed six gastric cancer cohorts from the TCGA and GEO databases. The results revealed that the enrichment scores of immune cells such as CD4^+^ T cells, CD8^+^ T cells, NKT cells and Th17 cells were significantly positively correlated with the HDS (Figure 4A). However, the scores of immunosuppressive cells such as M2 macrophages, MDSCs, pDCs and stromal cells (fibroblasts and endothelial cells) were significantly negatively correlated with the HDS. Interestingly, a significant positive correlation was found between the HDS and Treg cells (Figure 4A). In addition, we found that the HDS was considerably negatively correlated with the expression of angiogenic molecules in five gastric cancer cohorts (Figure 4B). The above studies revealed that a high HDS may indicate a “hot” tumor, but at the same time, these tumors exhibit immunosuppression.

However, the above results were based on public databases and mRNA expression. To further verify the accuracy of this result, we collected the cancer tissue of 121 patients with gastric cancer for RNA-seq and divided the patients into high and low HDS groups based on the median HDS (Figure 4C). Prognostic information was available for 41 patients with high HDSs and 45 patients with low HDSs. Survival analysis revealed that patients with a high HDS had a longer OS and DFS than those with a low HDS (Figure 4D). In addition, the correlation between the HDS and microenvironment cell enrichment scores was highly consistent with the results for five cohorts in the public database (Figure 4E). Results of multiple immunofluorescence staining revealed that a high HDS was associated with a higher proportion of CD8+ T-cell infiltration, but at the same time, tumor cells may express more PD-L1 to promote immune escape (Figure 4F). Immune checkpoint inhibitors could be used to obtain the greatest benefit in patients with a high HDS.

### The HDS is a powerful predictor of gastric cancer immunotherapy efficacy

**Figure 5:**
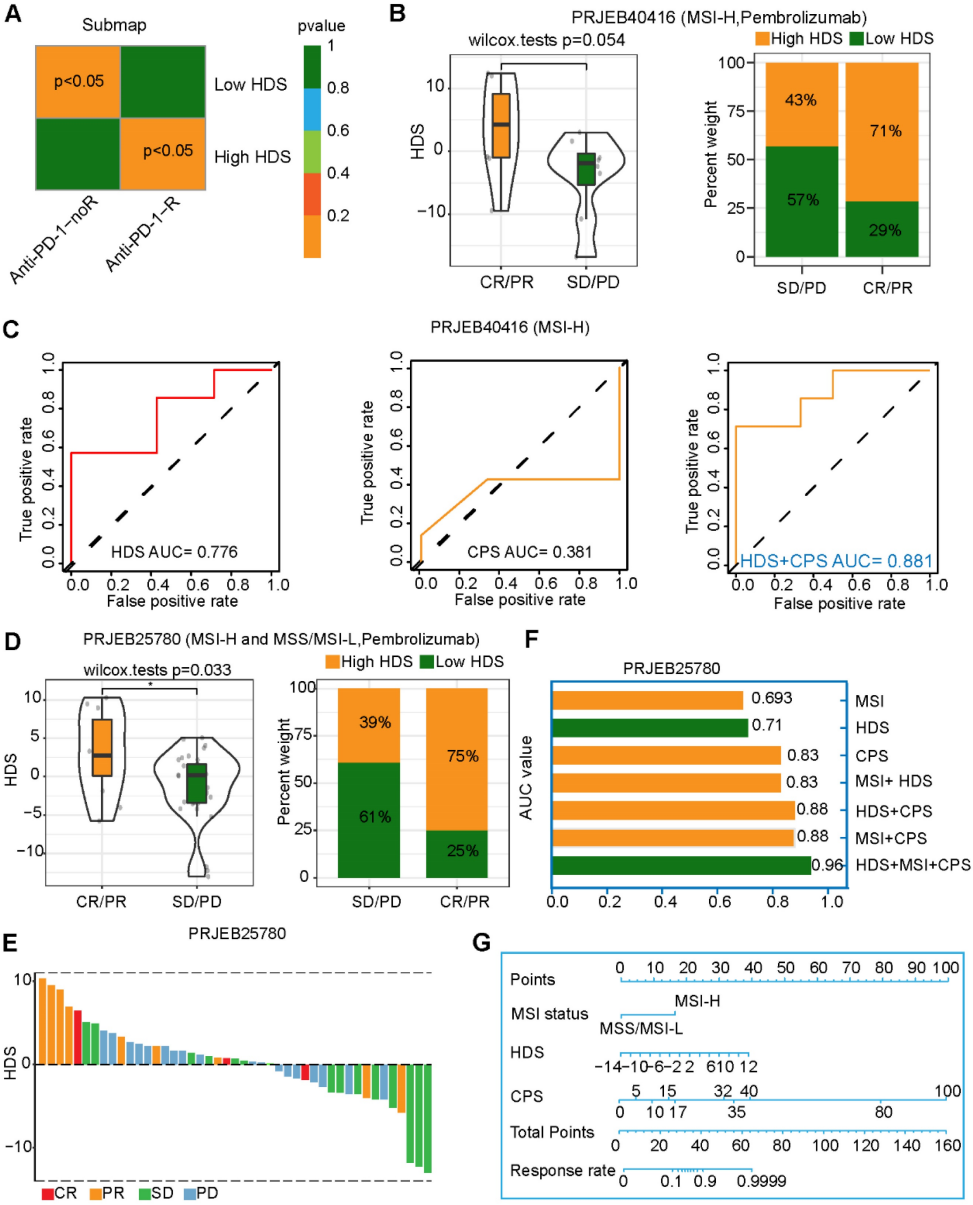
** Value of the HDS in predicting the efficacy of immunotherapy for gastric cancer patients. (A)** The TIDE algorithm and the submap algorithm predict the response of high- and low-HDS tumors to PD-1 inhibitors. **(B)** Differences in the HDS in different response groups (CR/PR and SD/PD) (PRJEB40416 cohort). **(C)** ROC curve reveals the accuracy of the HDS, CPS and HDS+CPS in predicting the efficacy of immunotherapy. **(D-E)** Differences in the HDS in different response groups (CR/PR and SD/PD) (PRJEB25780 cohort). **(F)** The AUC value of the ROC curve reveals the accuracy of different indicators for predicting the efficacy of immunotherapy. **(G)** The nomogram shows the HDS combined with MSI status and the CPS to predict the efficacy of gastric cancer immunotherapy.

The above results imply that patients with a high HDS are more likely to benefit from immunotherapy, as they have "hot" tumors. We also predicted that patients with a high HDS would benefit from PD-L1 inhibitor treatment through the TIDE and submap algorithms (Figure 5A). The study (PRJEB40416) included 14 patients with MSI-H advanced gastric cancer treated with pembrolizumab. The results revealed that the HDS was higher in the CR/PR group than in the SD/PD group, but the difference was not significant (*P* = 0.054) (Figure 5B). The reason for this could be the deviation caused by the small sample size. We observed that 71% of patients in the CR/PR group had a high HDS (Figure 5B). ROC curve analysis showed that the HDS predicted the therapeutic effect of pembrolizumab in the PRJEB40416 cohort with an AUC value of 0.776, while the AUC of the CPS was only 0.381, indicating that the HDS had a higher predictive value (Figure 5C). Finally, the HDS combined with the CPS was used to predict the efficacy of gastric cancer immunotherapy, with an AUC value of 0.881 (Figure 5C). To verify the reliability of the results, the study (PRJEB25780) included 45 patients with advanced gastric cancer treated with pembrolizumab. These patients included those with MSS, MSI-H, and EBV subtypes. In this cohort, CR/PR patients had a significantly higher HDS than SD/PD patients, with 75% of them having a high HDS (Figure 5D). The waterfall chart shows the HDS of each patient with different responses, and the ROC curve revealed an AUC value of 0.71 for the HDS in the PRJEB25780 cohort (Figure 5F). However, when combining the HDS with MSI status and the CPS, we found that the AUC of the combined model for predicting gastric cancer immunotherapy efficacy was as high as 0.96 (Figure 5F). This result was exciting and implied that the HDS combined with MSI status and the CPS could be used to accurately screen gastric cancer patients likely to benefit from immunotherapy. A nomogram was generated to visualize this predictive model (Figure 5G).

### Single-cell transcriptome sequencing revealed the TME of high and low HDS patients

**Figure 6:**
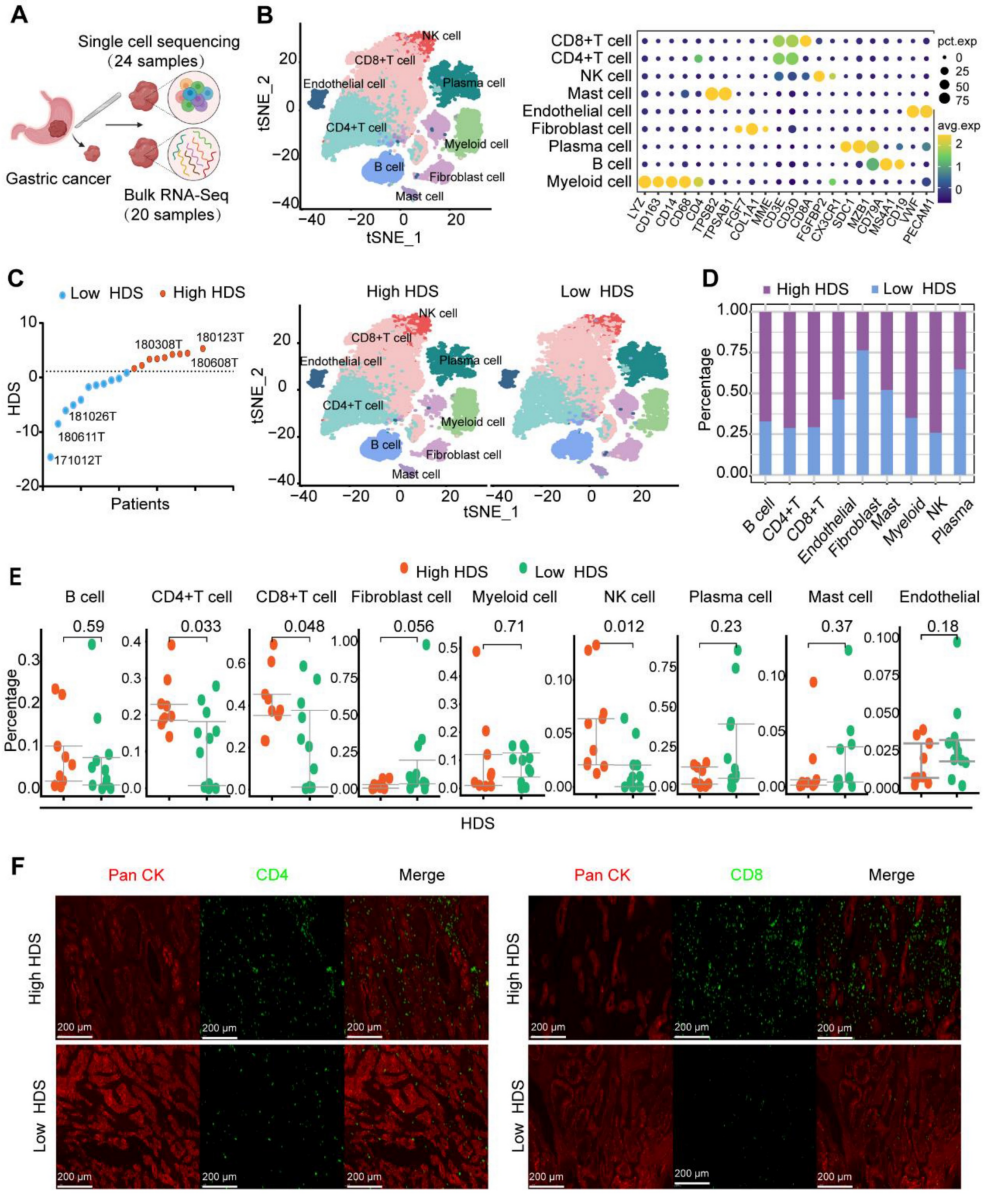
** Single-cell transcriptome sequencing reveals the TME of high- and low-HDS patients. (A)** Twenty-four single-cell sequencing datasets and twenty paired batch sequencing datasets were analyzed. **(B)** A total of 9 clusters were identified through T-distributed stochastic neighbor-embedding (t-SNE), according to the definition of marker genes. **(C)** The tSNE plot shows the characteristics of TME cells in patients with high and low HDSs. **(D)** Stacking plots revealed the proportion of high and low HDSs in each cell cluster. **(E)** Multiple immunofluorescence staining verified the difference in CD4^+^ T cells and CD8^+^ T cells in high- and low-HDS samples.

Based on the above results, a high HDS indicated a “hot” tumor, a better prognosis, and a better immunotherapy response. However, exploring the underlying factors in patients with high and low HDSs that lead to different immune characteristics may be an important strategy to improve immune infiltration and prolong survival. We used 24 single-cell sequencing datasets and 20 paired bulk sequencing datasets for analysis (Figure 6A). A total of 9 clusters were identified through T-distributed stochastic neighbor-embedding (t-SNE), according to the definition of marker genes as B cells (CD19, MS1A1), plasma cells (SDC1, MZB1, CD79A), CD4 T cells (CD4, CD3E, CD3D), CD8 T cells (CD8, CD3E, CD3D), natural killer (NK) cells (CX3CR1, FGFBP2), myeloid cells (CD68, CD163), mast cells (TPSAB1, TPSB2), endothelial cells (VWF, PECAM1), and fibroblasts (FGF7, COL1A1, MME) (Figure 6B). In addition, we separated the samples into high HDS and low HDS groups through bulk sequencing; there were 9 high HDS cases and 11 low HDS cases (Figure 6C). tSNE showed the characteristics of TME cells in patients with high and low HDS (Figure 6C). The stacking plots revealed that patients with a high HDS may have higher infiltration levels of CD8^+^ T cells, CD4^+^ T cells, and NK cells. Patients with a low HDS may have higher proportions of endothelial cells and fibroblasts (Figure 6D). Difference analysis further revealed that CD4^+^ T cells, CD8^+^ T cells and NK cells were more abundant in the high HDS group (Figure 6E). Multiple immunofluorescence experiments further verified these results (Figure 6F). These findings are consistent with our above analysis results based on bulk transcriptome sequencing.

### Endothelial cells and fibroblasts may inhibit the infiltration of T cells and NK cells through the MIF signaling pathway

**Figure 7:**
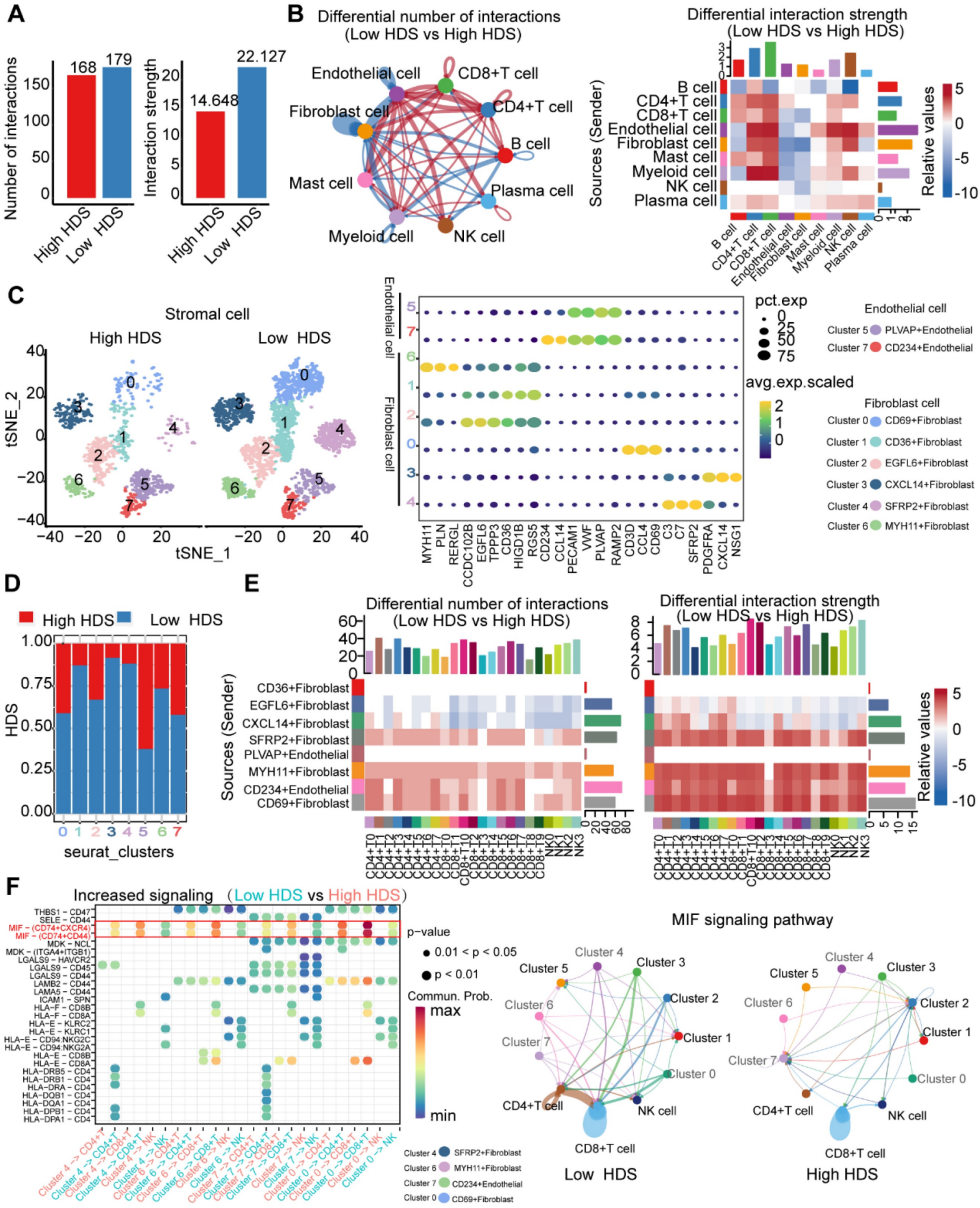
** Endothelial cells and fibroblasts may inhibit the infiltration of T cells and NK cells through the MIF signaling pathway. (A)** The difference in cell interaction number and strength in high- and low-HDS samples was analyzed based on CellChat. **(B)** Circle plot and heatmap show the difference in the number and intensity of interactions per cell cluster at high and low HDSs. **(C)** The tSNE analysis revealed reclustering maps of stromal cells. The bubble map shows the marker genes for each cell cluster. **(D)** Stacking plots show the proportion of high- and low-HDS samples in each cluster. **(E)** CellChat was used to analyze communication between endothelial cells, fibroblasts, T cells, and NK cells. **(F)** Bubble diagram and circle diagram showing that SFRP2^+^ fibroblasts, MYH11^+^ fibroblasts, CD234^+^ endothelial cells and CD69^+^ fibroblasts may communicate with T cells and NK cells frequently through MIF signals in low-HDS samples.

Next, we investigated the internal factors underlying TME differences between the high and low HDS samples. The CellChat package was used to compare cell interactions between the high HDS and low HDS groups. The results showed that there was no significant difference in the number of receptors or ligands between the two groups, and the interactions in the high HDS group were weaker than those in the low HDS group (Figure 7A). In the low HDS group, the number and strength of signaling pathways in endothelial cells, fibroblasts, and myeloid cells to CD4^+^ T cells, CD8^+^ T cells, and NK cells were significantly higher than those in the high HDS group (Figure 7B). We further reclustered endothelial cells and fibroblasts into eight cell clusters to explore the differences in stromal cells between high and low HDSs (Figure 7C). Each cluster's marker genes were identified using the FindMarkers function. There were two clusters of endothelial cells (PLVAP^+^ endothelial and CD234^+^ endothelial) and six clusters of fibroblasts (CD69^+^ fibroblasts, CD36^+^ fibroblasts, EGFL6^+^ fibroblasts, CXCL14^+^ fibroblasts, SFRP2^+^ fibroblasts, and MYH11^+^ fibroblasts). Stacking plots showed the proportion of high and low HDSs in each cluster (Figure 7D).

We further analyzed the communication information of endothelial cells and fibroblasts with T cells and NK cells. T cells and NK cells were reclustered into 7 clusters for CD4^+^ T cells (Figure S7), 11 clusters for CD8^+^ T cells (Figure S8), and 4 clusters for NK cells (Figure S9). CellChat was used to analyze communication between endothelial cells, fibroblasts, T cells, and NK cells. The results showed that the interactions between SFRP2^+^ fibroblasts, MYH11^+^ fibroblasts, CD234^+^ endothelial cells, CD69^+^ fibroblasts and T-cell and NK-cell clusters were stronger in the low HDS group (Figure 7E). We analyzed the differences in the communication of endothelial cells and fibroblasts (SFRP2^+^ fibroblasts, MYH11^+^ fibroblasts, CD234^+^ endothelial cells and CD69^+^ fibroblasts) with T cells and NK cells in the high and low HDS samples. The MIF signal was significantly enriched in the low HDS samples but not in the high HDS samples. The results suggested that SFRP2^+^ fibroblasts, MYH11+ fibroblasts, CD234^+^ endothelial cells and CD69^+^ fibroblasts may communicate with T cells and NK cells frequently through MIF signals, decreasing their infiltration levels in low HDS samples (Figure 7F). We further analyzed whether SFRP2^+^ fibroblasts, MYH11^+^ fibroblasts, CD234^+^ endothelial cells and CD69^+^ fibroblasts communicate with CD8^+^ T-cell subtypes, CD4^+^ T-cell subtypes and NK cell subtypes. It was also found that the MIF-CD74 signal was significantly enriched at a low HDS compared to a high HDS (Figure S10A).

However, the roles of SFRP2^+^ fibroblasts, MYH11^+^ fibroblasts, CD234+ endothelial cells and CD69^+^ fibroblasts are not clear in gastric cancer. The ssGSEA algorithm was used for enrichment analysis of each of the four cell clusters in the ACRG cohort. Survival analysis revealed that there were no significant associations between the expression of CD69^+^ fibroblasts and SFRP2^+^ fibroblasts and the survival of gastric cancer patients (Figure S11A). High levels of MYH11^+^ fibroblasts and CD234^+^ endothelial cells may be poor prognostic factors for patients with gastric cancer (Figure S11A). The top 20 marker genes of MYH11^+^ fibroblasts and CD234^+^ endothelial cells were analyzed by KEGG to identify their functions. MYH11^+^ fibroblasts were enriched in the NF-κB, epithelial-mesenchymal transition and IFN-γ signaling pathways, and CD234^+^ endothelial cells were enriched in the TGF-β, inflammatory reactions and IFN-γ signaling pathways (Figure S11B). These results suggest that MYH11^+^ fibroblasts and CD234^+^ endothelial cells may be widely involved in tumor and immune processes. Correlation analysis showed that MYH11^+^ fibroblasts and CD234^+^ endothelial cells were highly negatively correlated with the HDS (Figure S11 C). Furthermore, we found that assessing the HDS and MYH11^+^ fibroblasts could increase prognosis-based stratification accuracy in gastric cancer patients (Figure S11D).

These results further revealed that MYH11^+^ fibroblasts and CD234^+^ endothelial cells were the key factors affecting the difference in immune features between the high and low HDS groups. Inhibiting the infiltration of MYH11^+^ fibroblasts and CD234^+^ endothelial cells or blocking their inhibition of T cells and NK cells via MIF signals is an important strategy to enhance immune cell infiltration in low-HDS tumors.

### CCL17^+^ plasmacytoid dendritic cells may inhibit T-cell and NK-cell infiltration through the MIF signaling pathway in low-HDS tumors

**Figure 8:**
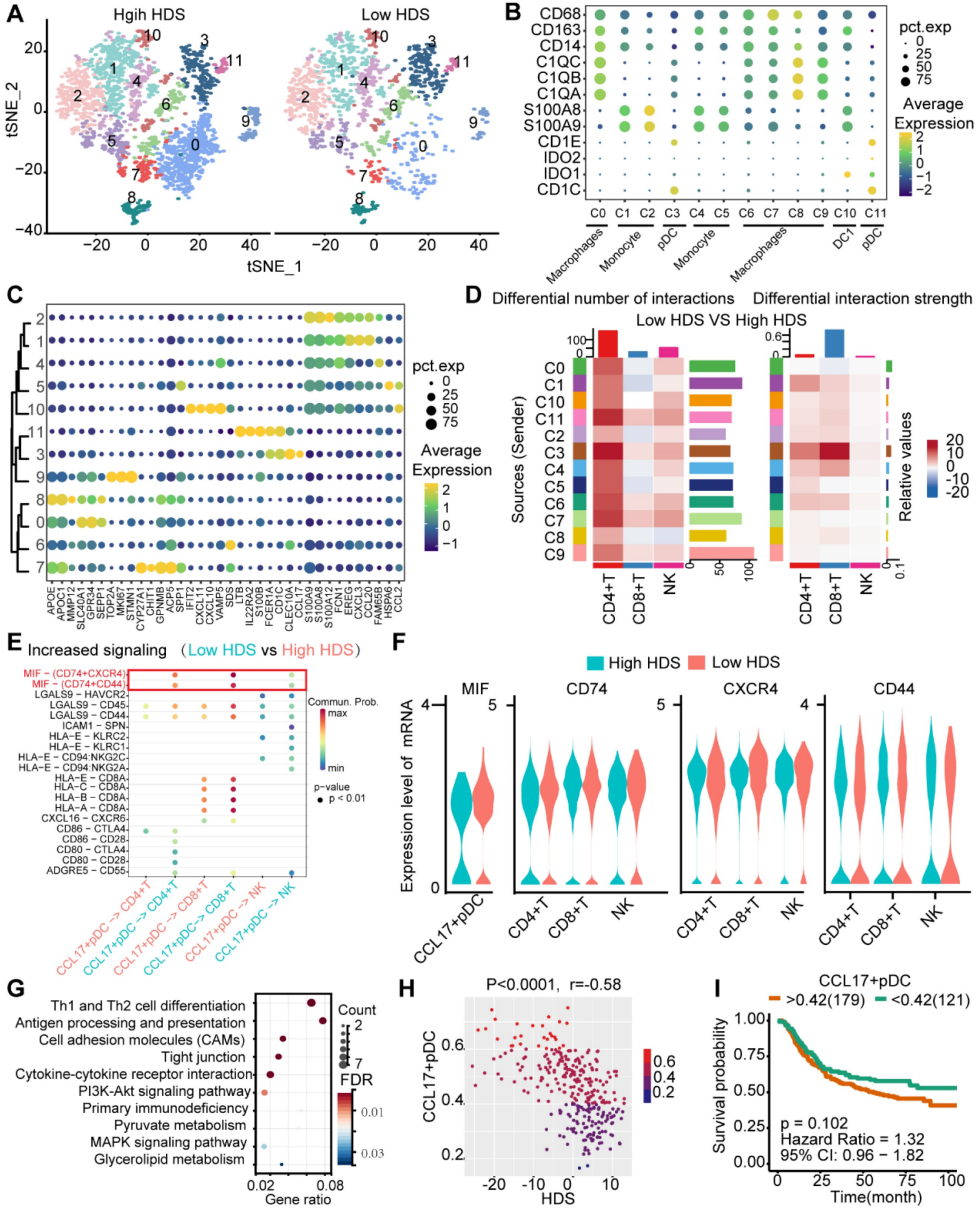
** CCL17^+^ plasmacytoid dendritic cells may inhibit T-cell and NK-cell infiltration through the MIF signaling pathway in low-HDS samples. (A)** The tSNE analysis revealed reclustering maps of myeloid cells. **(B)** The bubble plot shows the expression of the defined gene in each cell cluster. **(C)** The bubble plot shows the marker genes for each cell cluster. **(D)** The difference in cell interaction number and strength in high- and low-HDS samples was analyzed based on CellChat. **(E)** Bubble plot showing upregulated signaling pathways in low-HDS tumors. **(F)** Violin diagram showing the expression levels of MIF signaling pathway-associated ligands and receptors in CCL17^+^ pDCs, T cells, and NK cells. **(G)** KEGG demonstrates the capabilities of CCL17^+^ pDCs. **(H)** Correlation between the enrichment score of CCL17^+^ pDCs and the HDS (ACRG cohort). **(I)** Effect of the enrichment score of CCL17^+^ pDCs on the prognosis of gastric cancer (ACRG cohort).

Previous results found that myeloid cells also communicate with T cells and NK cells in low HDS tumors (Figure 7B). We reclustered the myeloid cells into 12 cell clusters (Figure 8A). According to the definition of marker genes, macrophages (CD163, CD68), monocytes (S100A8, S100A9), plasmacytoid dendritic cells (pDCs) (CD1E, CD1C), and DC1 cells (IDO1, IDO2) were used (Figure 8B). Using the FindMarkers function, each cluster's marker genes were identified. The top 5 marker genes of each cluster are shown in the bubble plot (Figure 8C). Cell communication analysis revealed that cluster 3 (C3) cells communicated more frequently with T cells and NK cells in low-HDS tumors (Figure 8D). C3 cells were identified as CCL17^+^ pDCs based on marker gene characteristics (Figure 8B-C).

By comparing the differentially enriched signaling pathways between low- and high-HDS tumors, we again found that CCL17^+^ pDCs frequently communicate with T cells and NK cells through MIF signaling in low-HDS tumors (Figure 8E). We further analyzed the ligands and receptors associated with the MIF signaling pathway in CCL17^+^ pDCs, T cells and NK cells. The results showed that MIF expression in CCL17^+^ pDCs was higher in low-HDS tumors. The expression levels of MIF signaling pathway-associated receptors (CD74, CXCR4, CD44) were not different between T cells and NK cells in high- and low-HDS tumors (Figure 8F). Similarly, we found that the MIF-CD74 signal was significantly enriched in low-HDS tumors in the cellular communication network between CCL17^+^ pDCs and CD8^+^ T-cell subtypes, CD4^+^ T-cell subtypes, and NK-cell subtypes (Figure S10B). KEGG enrichment analysis of marker genes revealed that CCL17^+^ pDCs were mainly enriched in Th1 and Th2 cell differentiation, antigen presentation and cell adhesion pathways (Figure 8G). Correlation analysis showed that the enrichment score of CCL17^+^ pDCs in the ACRG cohort was negatively correlated with the HDS (Figure 8H). However, the CCL17^+^ pDC score had no significant effect on prognosis (Figure 8I). These results suggest that in low-HDS tumors, CCL17^+^ pDCs may communicate with T cells and NK cells frequently through high MIF expression. Blocking the communication network of MIFs may enhance T-cell and NK-cell infiltration.

**Figure 9:**
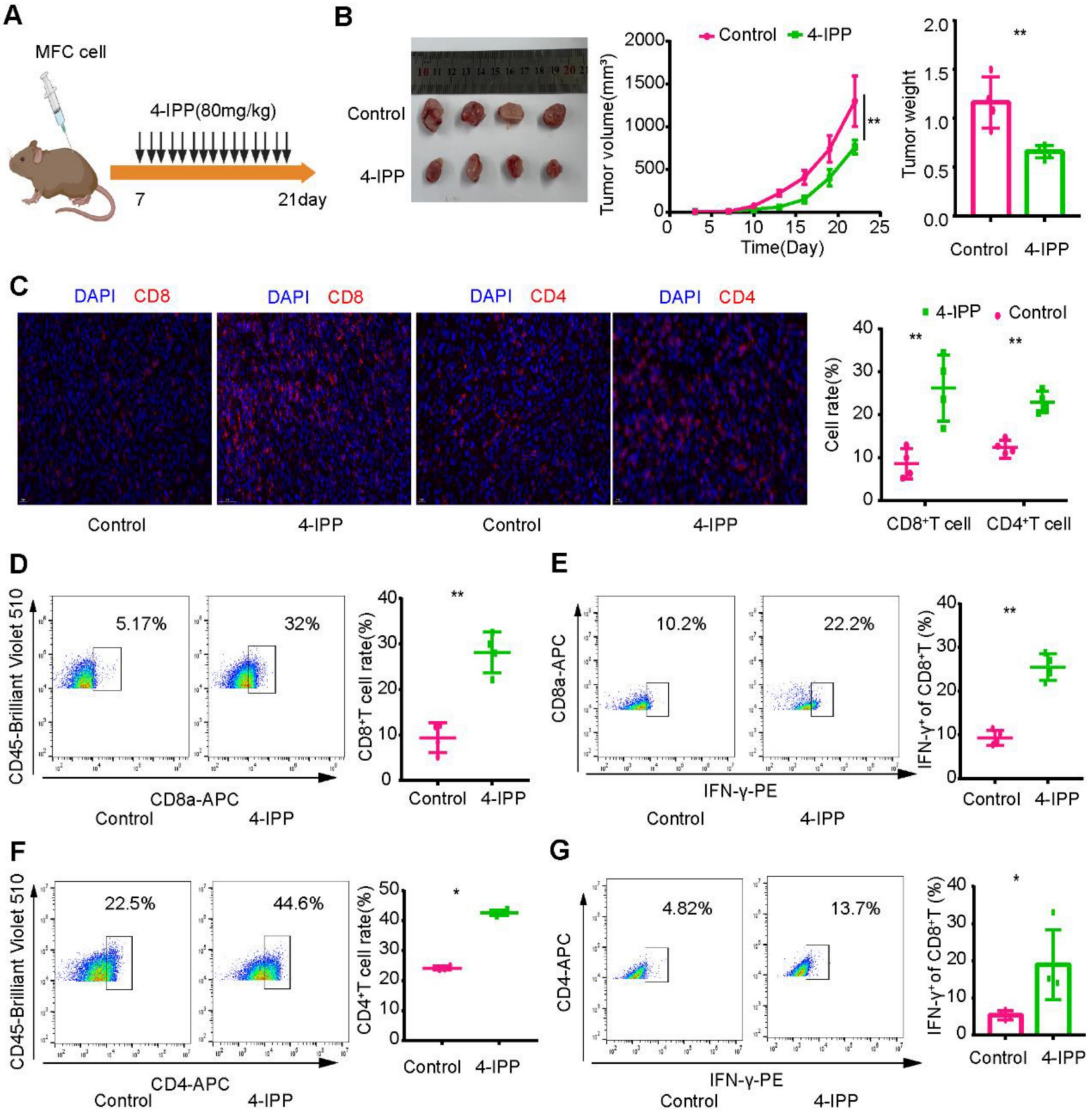
** Inhibition of MIF signaling enhances intratumoral T-cell infiltration and cytotoxicity. (A)** Regimen of MIF inhibitors for gastric cancer. **(B)** MIF inhibitors significantly inhibited the growth of gastric cancer. **(C)** Immunofluorescence revealed the infiltration level of T cells in the MIF-inhibited group and the control group. **(D-E)** Flow cytometry was used to analyze the proportion and cytotoxicity of CD8^+^ T cells in the MIF inhibitor and control groups. **(F-G)** Flow cytometry was used to analyze the proportion and cytotoxicity of CD4^+^ T cells in the MIF inhibitor and control groups. *P < 0.05, **P < 0.01.

To verify these results, we constructed a mouse model of heterotopic transplanted tumors treated with intraperitoneal injection of an MIF inhibitor (4-IPP) (Figure 9A). The results showed that MIF inhibitors significantly inhibited tumor growth (Figure 9B). Immunofluorescence revealed that CD8^+^ T-cell and CD4^+^ T-cell infiltration increased significantly after MIF inhibitor treatment (Figure 9C). Flow cytometry analysis revealed that the proportion of CD8^+^ T cells and CD4^+^ T cells in lymphocytes and their capability to secrete IFN-γ increased significantly after MIF inhibitor treatment (Figure 9D-G). The above results further verified the results of single-cell transcription analysis. By inhibiting MIF signaling, the TME characteristics of gastric cancer may be reversed, leading to the transformation of cold tumors into hot tumors.

### GPX4 may be an important target for tumor cells in low-HDS tumors

**Figure 10:**
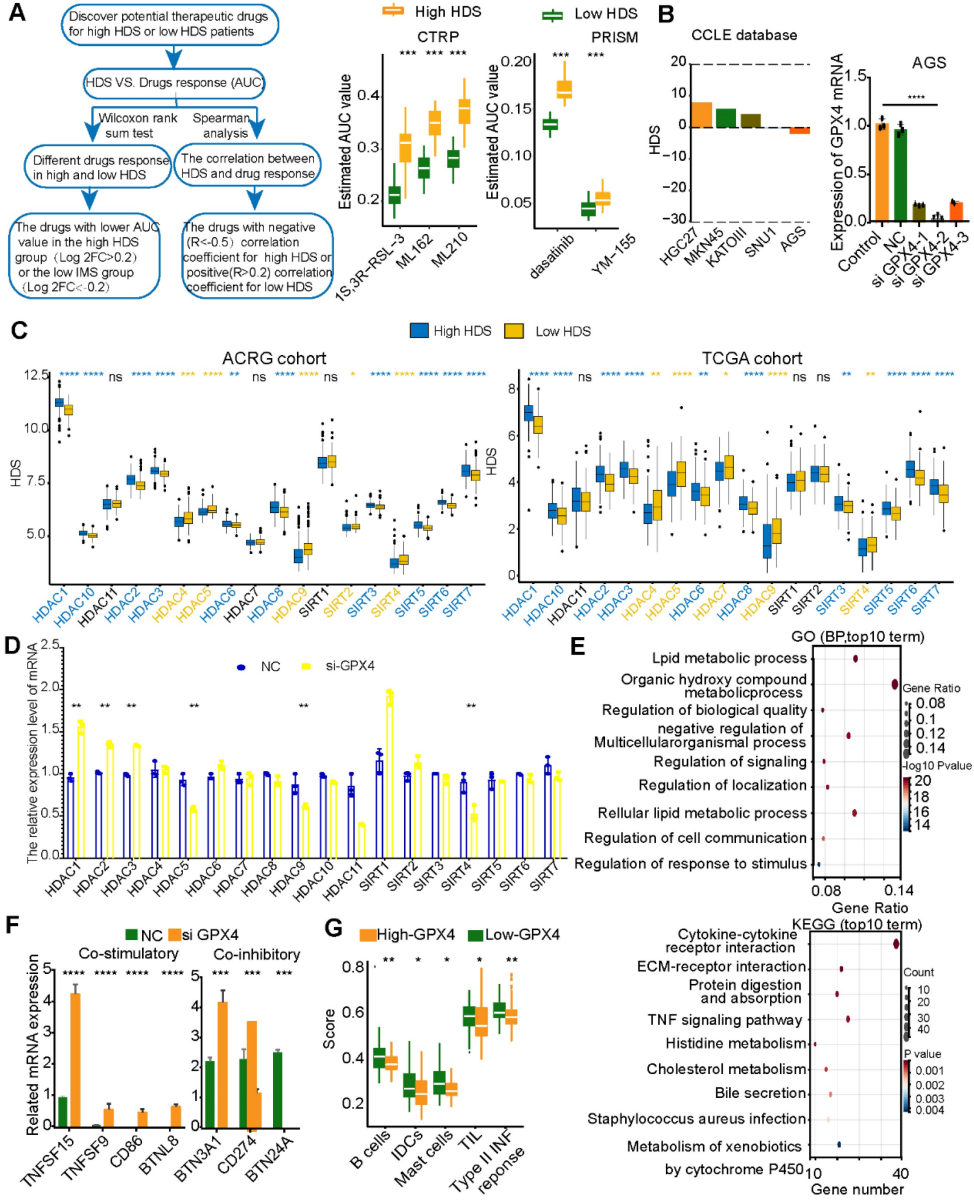
** Inhibition of GPX4 expression in tumor cells may activate the immune microenvironment. (A)** The CTRP and PRISM databases predicted effective compounds for low-HDS samples. **(B)** HDS values for five gastric cancer cell lines. GPX4 mRNA expression in different intervention groups. **(C)** Differential expression of HDACs in the high- and low-HDS groups. **(D)** Expression of HDACs after knocking down GPX4. **(E)** GO and KEGG functional enrichment analyses of the differentially expressed genes in the NC group and the GPX4 knockdown group. **(F)** Differential analysis of the expression of costimulatory and coinhibitory molecules after GPX4 knockdown. **(G)** Differential analysis of immune cell infiltration and immune-related signatures in the high- and low-HDS groups. *P < 0.05, **P < 0.01, ***P < 0.001, ****P < 0.0001.

Based on the above results, blocking MIF may enhance immune infiltration in low-HDS patients by inhibiting the effect of stromal cells and CCL17^+^ pDCs on T cells and NK cells. This strategy is realized by interfering with cell communication in the TME. However, due to the lack of tumor cells in single-cell transcriptome sequencing data, it is impossible to mine tumor cell targets to improve patient prognosis. For this reason, the CTPR and PRISM databases were used to identify chemotherapy drugs that might be effective against high-HDS and low-HDS tumors (Figure 10A). We found that gastric cancer tumors with a low HDS may be sensitive to GPX4 inhibitors (ML210, ML162, and 1S3R-RSL-3), survivin inhibitors (YM-155), and dasatinib (Figure 10A). Three GPX4 inhibitors were predicted to be more effective against low-HDS tumors; therefore, it was necessary to investigate the regulatory mechanism of GPX4 inhibitors in patients with low-HDS tumors. To explore the effect of GPX4 inhibitors on gastric cancer tumors with a low HDS, we used five types of gastric cancer cells (HGC27, MKN45, KATOAIII, SNU1, and AGS) stored in the laboratory. The CCLE database was used to analyze the HDS value of the cells, and it was found that AGS cells had the lowest HDS (Figure 10B); thus, AGS cells were used in our analysis. Transfection of three si-GPX4s into AGS cells showed that siGPX4-2 had a significant knockdown effect on GPX4 expression (Figure 10B). Table S6 shows the primer sequences of all genes. To explore why GPX4 inhibitors are more effective against tumors with a low HDS, we analyzed the expression characteristics of HDACs in patients with a high and low HDS. The results showed that people with a high HDS may have higher HDAC1, HDAC2, HDAC3, HDAC6, HDAC8, HDAC10, SIRT5, SIRT6 and SIRT7 expression levels. Patients with a low HDS had higher expression levels of HDAC4, HDAC5, HDAC9, SIRT2, and SIRT4 (Figure 10C). Therefore, we further found by RT‒PCR that GPX4 knockdown significantly inhibited the expression of HDAC5, HDAC9 and SIRT4 and enhanced the expression of HDAC1, HDAC2 and HDAC3 (Figure 10D). This may be one of the reasons why low-HDS tumors were more sensitive to GPX4 inhibitors.

Next, RNA-seq was used to explore the effect of GPX4 knockdown. Differentially expressed genes in the NC and siGPX4 groups were analyzed using GO and KEGG functional enrichment analyses. The results revealed that these differentially expressed genes were mainly enriched in cell communication and metabolic processes. KEGG enrichment analysis revealed that after GPX4 knockdown, metabolic pathways and immune-related signaling pathways (TNF signaling pathway and cytokine‒cytokine receptor pathway) were activated (Figure 10E). These results suggest that GPX4 knockdown may regulate cell metabolism and improve tumor cell immunogenicity. We further analyzed the expression of costimulatory and coinhibitory molecules. We found that the costimulatory molecules TNFSF15 and TNFSF9 were overexpressed in the siGPX4 group (Figure 10F), suggesting that GPX4 knockdown could inhibit tumor angiogenesis. We also found that the mRNAs of CD86 and BTNL8 were expressed in the siGPX4 group (Figure 10F), suggesting that knocking down GPX4 increased T-cell activity and killed tumor cells. In addition, T-cell activity was related to the expression of immune checkpoint molecules. We also found that PD-L1 mRNA expression was significantly reduced in the siGPX4 group (Figure 10E), suggesting that GPX4 knockdown could inhibit PD-L1 expression and prevent tumor immune escape. However, GPX4 knockdown significantly increased the mRNA levels of BNT3A1 and BNT24A (Figure 10F), which may act as a negative feedback regulatory mechanism on T-cell activity. Finally, the ACRG cohort was used to analyze immune cell expression and related immune signals in the high- and low-GPX4 groups (Figure 10G). The results showed that GPX4 knockdown significantly increased the infiltration of B cells, iDCs and mast cells, the level of TILs and the expression of type II TNF response-related factors. The results above suggest that GPX4 knockdown may activate T-cell activity, leading to enhanced TIL infiltration and the type II TNF response to kill tumor cells.

### GPX4 knockdown may promote CD8^+^ T-cell infiltration and improve the anti-PD-L1 therapeutic effect

**Figure 11:**
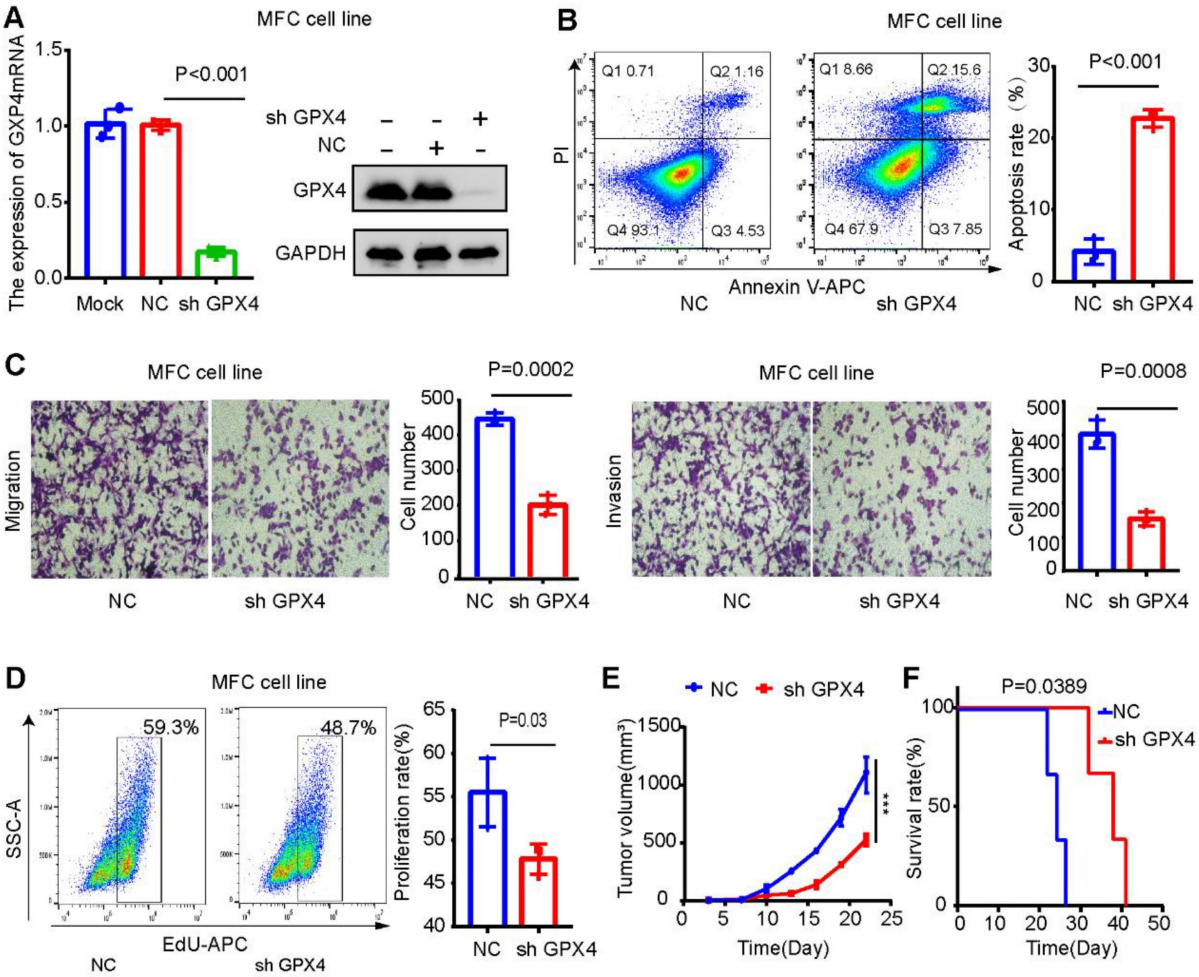
** GPX4 knockdown inhibits tumor growth and liver metastasis in gastric cancer. (A)** Western blot and qPCR assays verified the knockdown efficiency of GPX4 in MFC cells. **(B)** The effect of GPX4 knockdown on cell apoptosis was detected by apoptosis assay. **(C)** The effect of GPX4 knockdown on cell migration and invasion was detected by cell migration and invasion assays. **(D)** The EdU assay was used to detect the effect of GPX4 knockdown on cell proliferation. **(E-F)** The mouse subcutaneous xenograft model revealed that knockdown of GPX4 significantly inhibited tumor cell growth and prolonged the survival time of mice. **P < 0.01.

GPX4 expression was downregulated in MCF cells (mouse gastric cancer cells) to explore its effect on the gastric cancer microenvironment. The results were verified by Western blot and qPCR assays (Figure 11 A). GPX4 knockdown significantly promoted apoptosis and inhibited the migration, invasion, and proliferation of MFC cells (Figure 11B-D). The mouse xenograft model revealed that GPX4 knockdown inhibited tumor cell growth and prolonged the survival time of mice (Figure 11E-F).

**Figure 12:**
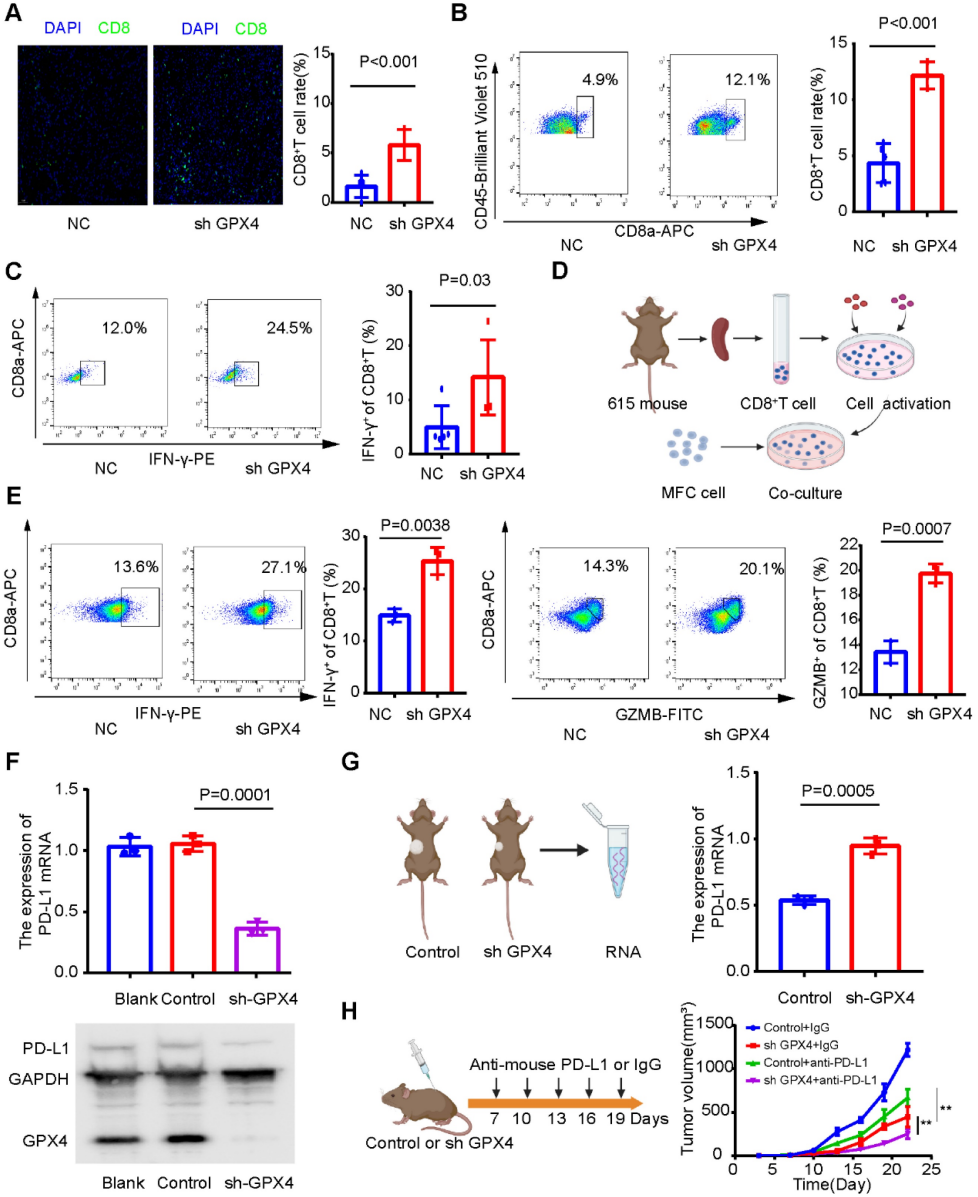
** GPX4 knockdown may promote CD8**^+^** T-cell infiltration and cytotoxicity**. **(A)** Immunofluorescence assay showed that GPX4 knockdown significantly promoted the infiltration level of CD8^+^ T cells. **(B)** Flow cytometry was used to analyze the infiltration level of CD8^+^ T cells in tumor tissues after knockdown of GPX4. **(C)** Flow cytometry showed that GPX4 knockdown significantly promoted IFN-γ secretion by CD8^+^ T cells and increased the toxicity of CD8^+^ T cells. **(D)** Activation of CD8^+^ T cells and coculture with tumor cells. **(E)** The cytotoxicity of CD8^+^ T cells after coculture was determined by flow cytometry. **(F)** GPX4 knockdown significantly inhibited the expression of PD-L1 mRNA and protein. **(G)** Expression level of PD-L1 mRNA in mouse subcutaneous tumors. **(H)** GPX4 knockdown combined with PD-L1 inhibitors significantly improved the efficacy of immunotherapy and inhibited tumor growth. *P < 0.05, **P < 0.01.

Next, we explored whether GPX4 knockdown plays a tumor suppressor role by regulating the immune microenvironment. Immunofluorescence assays showed that GPX4 knockdown increased CD8^+^ T-cell infiltration (Figure 12A), and flow cytometry of subcutaneous tumor tissue confirmed this result (Figure 12B). In addition, CD8^+^ T cells in subcutaneous tumors with low GPX4 had stronger IFN-γ secretion ability (Figure 12C). These results revealed that GPX4 knockdown promoted CD8^+^ T-cell infiltration. However, it is unclear whether tumor cells affect CD8^+^ T cells, directly or indirectly, through other immune cells after GPX4 knockdown. To explore this result, 615 mouse spleen cells were extracted, and CD8^+^ T cells were sorted by magnetic bead separation and then activated *in vitro* (Figure 12D). Activated CD8^+^ T cells were cocultured with MFC-NC and MFC-SH GPX4 cells. Before cell collection, brefeldin A was applied to block cytokine secretion. Flow cytometry showed that GPX4 knockdown promoted IFN-γ and GZMB secretion by CD8^+^ T cells and increased CD8^+^ T-cell toxicity (Figure 12E). These results indicated that GPX4 knockdown not only inhibited tumor cell growth but also improved CD8^+^ T-cell infiltration and killing ability.

However, the mechanism by which GPX4 affects the infiltration level and killing ability of CD8^+^ T cells remains unclear. Our above results showed that GPX4 knockdown significantly inhibited PD-L1 mRNA expression in AGS cells (Figure 10E), so we speculated that GPX4 knockdown might inhibit PD-L1 expression and affect CD8^+^ T-cell infiltration in tumors. Interestingly, GPX4 knockdown significantly inhibited PD-L1 expression in MFC cells (Figure 12F), while an increase in PD-L1 expression was observed in the subcutaneous tumor model (Figure 12G). We speculated that GPX4 knockdown enhanced CD8^+^ T-cell infiltration, which might lead to PD-L1 overexpression by other immunosuppressive cells to inhibit CD8^+^ T cells. Based on this result, we found that knockdown of GPX4 combined with PD-L1 inhibitor therapy can improve immunotherapy efficacy in gastric cancer (Figure 12H), suggesting a new therapeutic strategy for this disease.

## Discussion

Studies have shown that HDAC regulators play an indispensable role in regulating the TME 17, 38. Most previous studies have explored the function of a single HDAC or a class of HDACs, but different HDACs play highly heterogeneous roles in regulating the TME and antitumor effects 38-40. Therefore, our study reveals the mechanism underlying TME differences and provides a new therapeutic strategy for gastric cancer by comprehensively analyzing HDAC-related TME features and quantifying TME indicators.

In this study, we performed multiomics analysis of HDACs in gastric cancer, including analysis of gene mutations, somatic CNVs, and transcriptome expression levels. We found that different HDACs have certain genomic characteristics, even within the same class of HDACs. To explore whether HDAC expression is related to the TME, ssGSEA and correlation analysis were performed. We found that HDAC expression was significantly correlated with cell infiltration in the TME (Figure 1B). However, the correlations of HDACs with TME cell infiltration levels were highly heterogeneous. For example, a significant positive correlation was found between HDAC9 expression and cell infiltration in the TME, which is consistent with previous reports that HDAC9 could activate the innate immune system and regulate Treg cells 41. In addition, we found that the high expression of HDAC11 may cause tumor cell immune desertion and inhibit cell infiltration in the TME. This is consistent with a report showing that HDAC11 is a negative regulator of the inflammatory T-cell response and that HDAC11 deficiency leads to a weaker inhibitory response to regulatory T cells 42, 43. These results indicate that HDACs play a critical role in the TME and regulate cell infiltration in a heterogeneous manner. Expression of a single HDAC may not be sufficient for accurately assessing the TME status. Therefore, exploring the overall HDAC expression pattern will help obtain a deeper understanding of the gastric cancer TME.

In this study, we identified three different HDAC subtypes based on the expression profile characteristics of HDACs. HDAC cluster A had the worst prognosis, while cluster C had the best. To explore the characteristics of different HDAC clusters, we identified signaling pathways between certain clusters through gene enrichment analysis. In this study, we found that HDAC cluster A activated EMT signals, and this activation in gastric cancer could be related to a poor prognosis 44, 45. HDAC cluster C was enriched in antigen presentation and CD8^+^ T-cell receptor activation and highly enriched in the mismatch repair pathway, suggesting that HDAC cluster C was related to stronger immune cell infiltration and immunogenicity 46. Researchers have reported that solid tumors can be divided into immunoinflammatory types (“hot” tumors) and immune exclusion types (“cold” tumors) based on TME characteristics 47-49. According to this study, different HDAC clusters were significantly related to cell infiltration levels in the immune microenvironment of gastric cancer. Cluster C was extensively enriched in activated T cells, DCs, and other immunosuppressive cells, while cluster A had a high infiltration level of immunosuppressive cells, such as MDSCs and regulatory T cells (Figure S4). This indicates that HDAC cluster C may be related to the characteristics of “hot” tumors and that patients with this subtype may benefit more from immunotherapy than those with other subtypes.

Although HDAC expression is significantly correlated with TME cell infiltration and the identification of TME subtype characteristics in gastric cancer (Figure 1 and Figure S2), it is difficult to quantify TME indicators based on HDAC clusters. We analyzed the gene characteristics between different HDAC clusters, and PCA and the Boruta algorithm were used to construct the HDS model to evaluate the HDAC cluster characteristics and quantify the TME of gastric cancer. Based on the median value, we divided gastric cancer samples into high- and low-HDS groups, and the high-HDS group had a better prognosis (Figure 3). Studies have revealed that MSI tumors have a high mutation burden and respond well to immune checkpoint inhibitor therapy 50, 51, and EBV-positive tumors are characterized by a significant infiltration of immune cells 10, 52. Matrix activation in EMT and gastric cancer subtypes has been identified as the key driver of checkpoint inhibitor therapy failure 53. Our research showed that the HDS significantly differed among different gastric cancer subtypes. The HDS was highest in the MSI-H and EBV subtypes and lowest in the EMT and GS subtypes (Figure 3). These findings suggest that the HDS could effectively stratify gastric cancer subtypes and has a close connection with the gastric cancer TME.

Furthermore, we explored whether the HDS could be used as an indicator to quantify the TME. There is evidence that the expression characteristics of antigen-presenting molecules, costimulatory molecules, and coinhibitory molecules can reflect tumor immunogenicity and immune escape 50, 54. We found that the HDS was positively correlated with antigen-presenting molecules and costimulatory molecules (Figure 3F). To examine the relationship between the HDS and TME, we analyzed six gastric cancer cohorts in the TCGA and GEO databases (Figure 4A). A significant positive correlation was found between the HDS and the enrichment scores of immune cells, including activated CD4^+^ T cells, activated CD8^+^ T cells, NKT cells and TH17 cells. However, immunosuppressive cells such as M2 macrophages, MDSCs, pDCs, and stromal cells (fibroblasts and endothelial cells) were negatively correlated with the HDS. Interestingly, we found that the HDS was positively correlated with the enrichment score for Treg cells. This result suggests that patients with gastric cancer with a high HDS have stronger immunogenicity; however, those with a high HDS have higher expression levels of cosuppressive molecules and may have stronger immune escape. This result was verified through RNA-seq and immunohistochemical analysis, which showed that patients with a high HDS had significant CD8^+^ T-cell infiltration and high PD-L1 expression. High PD-L1 expression has been proven to be the means by which tumor cells evade immune cell-mediated killing 55-58. These findings suggest that patients with a high HDS may benefit from immune checkpoint inhibitor treatment.

To explore whether a high HDS could be used to predict immunotherapy efficacy, we analyzed two cohorts of patients treated with pembrolizumab for advanced gastric cancer (Figure 5). In the PRJEB40416 cohort, the HDS-predicted immunotherapy effect achieved an AUC of 0.776, while the CPS was unable to do so (AUC = 0.381). In the PRJEB25780 cohort, the same HDS model for predicting immunotherapy efficacy had an AUC of 0.71. However, when we combined the MSI status and CPS, we found that the predictive efficacy was as high as 0.96. This result suggests that the assessment of the HDS combined with traditional immunotherapy efficacy indicators could significantly increase the predictive power. The above findings reveal that patients with high-HDS gastric cancer have a better prognosis and can benefit from immunotherapy. However, for patients with low-HDS gastric cancer (“cold” tumors), the development of a treatment strategy is difficult. Therefore, it is necessary to decipher the key factors underlying the immunosuppressive state of low-HDS tumors.

Single-cell transcriptome sequencing has been shown to be an important method for characterizing the TME 59. Therefore, we further identified the reason for the intrinsic TME difference between high- and low-HDS tumors by single-cell transcriptome sequencing analysis. Endothelial cells and fibroblasts play key roles in promoting tumor immunosuppressive microenvironments 21, 60, 61. Through single-cell sequencing analysis, we found that the frequency and intensity of communication between endothelial cells and fibroblasts with T cells and NK cells in the low-HDS group was much higher than that in the high-HDS group (Figure 7B). By reclustering stromal cells, we found that SFRP2^+^ fibroblasts, MYH11^+^ fibroblasts, CD234^+^ endothelial cells, and CD69^+^ fibroblasts may frequently communicate with T and NK cells in low-HDS tumors by MIF signaling. However, it was not observed in high-HDS tumors (Figure 7F). Cancer-associated fibroblasts provide an immunosuppressive microenvironment via MIF signaling 62. MIF-CD74 signaling was found to promote the production of lactic acid and inhibit the activation of T cells 63. Blocking MIF signaling may enhance CD8^+^ T-cell and CD4^+^ T-cell infiltration and improve immunotherapy efficacy 63. Blocking their inhibition of T cells and NK cells via MIF signals is an important strategy to enhance immune cell infiltration in patients with low-HDS tumors. Tumor-infiltrating pDCs reduce IFN-α production and promote the expansion of Tregs, thus contributing to tumor immune tolerance and progression 64. Melanoma pDCs express indoleamine 2,3-dioxygenase, which consumes tryptophan and leads to T-cell impotence and immune tolerance 65. This evidence suggests that pDCs induce immunosuppressive immune responses. In this study, we found that CCL17^+^ pDCs frequently communicate with T cells and NK cells through MIF signaling in low-HDS tumors (Figure 8E). We further analyzed the ligands and receptors associated with the MIF signaling pathway in CCL17^+^ pDCs, T cells and NK cells. The results showed that the expression of MIF in CCL17^+^ pDCs in tumors with a low HDS was higher than that in CCL17^+^ pDCs in tumors with a high HDS. These results suggest that endothelial cells, fibroblasts, and pDCs may frequently communicate with T cells and NK cells through high expression of MIF in low-HDS tumors. Finally, we tested the mouse subcutaneous tumor model and found that blocking the communication network of MIFs may contribute to increasing the infiltration level of T cells in the TME of low-HDS tumors. However, further animal studies may be needed to confirm which cell type plays a more profound role in the MIF-regulated signaling pathway, which is also a limitation of this study.

Due to the lack of tumor cells in single-cell transcriptome sequencing data, it is impossible to mine tumor cell targets to improve patient prognosis. The CTRP and PRISM databases can be combined with the CCLE database to explore therapeutic targets for different cancer types based on the gene expression characteristics of tumor cell lines 35, 36. Through algorithm analyses, we found that GPX4 inhibitors were more effective in patients with low-HDS tumors than in those with high-HDS tumors (Figure 10A). GPX4 is the key protein for ferroptosis; it induces lipid peroxidation, inhibits ferroptosis, and inhibits the infiltration of TME cells 66. We knocked down GPX4 expression in AGS cells and found that multiple metabolic and immune signaling pathways were activated. In addition, the expression levels of costimulatory molecules also increased, while the expression level of PD-L1 mRNA was significantly reduced. This implies that GPX4 knockdown may enhance tumor cell immunogenicity and prevent tumor cells from escaping immune surveillance. In *in vivo* and *in vitro* studies, GPX4 expression in MFC cells was downregulated, which significantly enhanced CD8^+^ T-cell infiltration and cytotoxicity. GPX4 knockdown combined with PD-L1 inhibitors significantly enhanced immunotherapy efficacy (Figure 12). This provides a theoretical basis for improving tumor immunogenicity and formulating treatment strategies for patients with low-HDS tumors.

In conclusion, this study revealed that the HDS could accurately reflect the subtype characteristics and provide a quantitative measure of the TME in gastric cancer. A high HDS may indicate a “hot” tumor that can benefit from immunotherapy. In addition, a low HDS may activate the immune microenvironment by inhibiting the MIF signaling pathway in the TME and regulating GPX4 expression in tumor cells. We believe these findings will contribute to discovering new therapeutic targets and developing effective treatment strategies for gastric cancer.

## Supplementary Material

Supplementary figures and tables.

## Abbreviations

HDACs: Histone deacetylases
HDS: HDACs score
TME: Tumor microenvironment
SCNV: Somatic copy number variation
GEO: Gene Expression Omnibus
NMF: Nonnegative matrix factorization
CPS: combined positive score
PCA: principal component analysis
