# Supplementary material for: Histone deacetylase-mediated tumor microenvironment characteristics and synergistic immunotherapy in gastric cancer
Source: Theranostics. 2023 Aug 18;13(13):4574–600. doi: 10.7150/thno.86928 (PMC10465215; doi:10.7150/thno.86928)
Supplement: Supplementary file 1 — Supplementary figures and tables. [file thnov13p4574s1.zip › Supplementary figures.pdf]

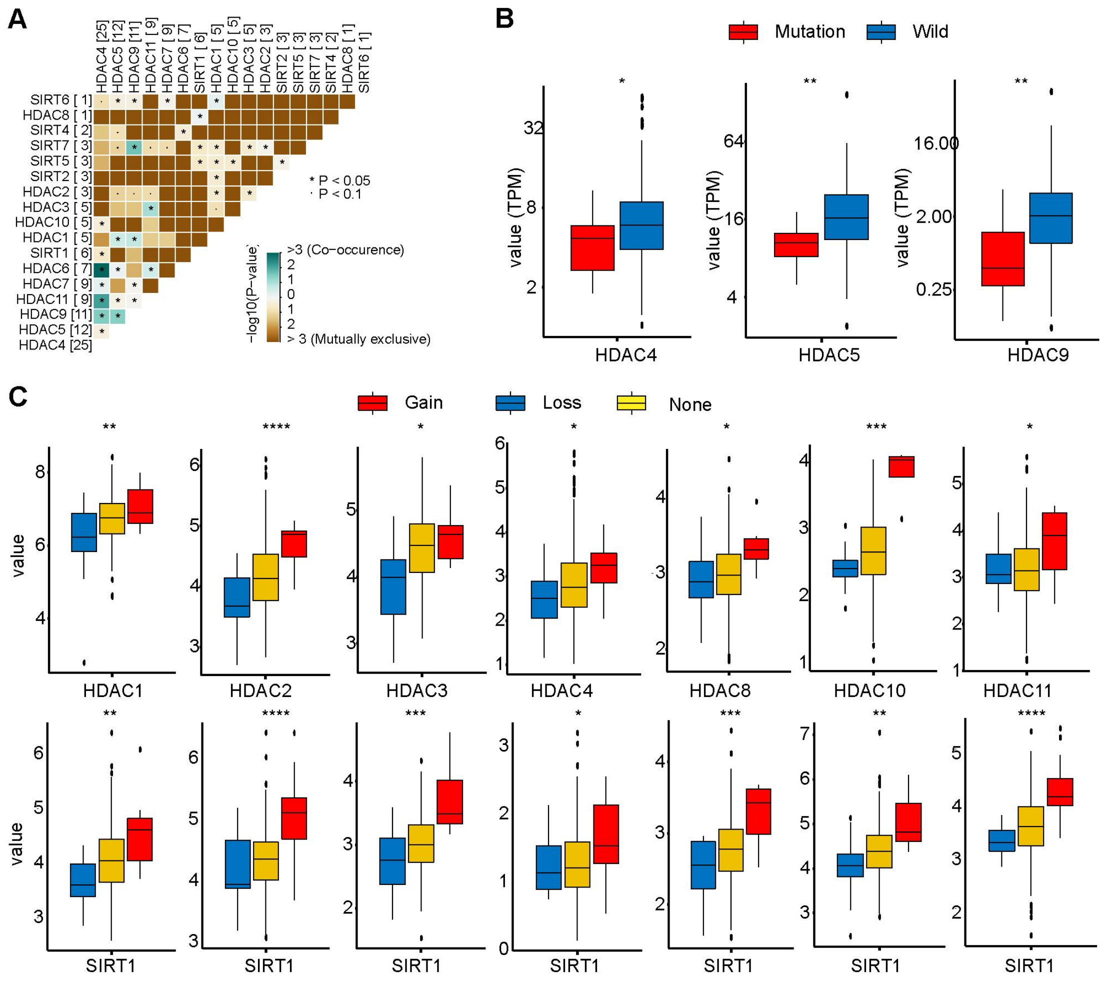

Figure S1 The relationship between HDAC mRNA expression and epigenetic characteristics

(**A**) Mutations in HDACs. (**B**) The relationship between HDAC mutations and mRNA expression. (**C**) The relationship between copy number variation of HDACs and mRNA expression. * P<0.05, **P<0.01, ***P<0.001, ****P<0.0001.

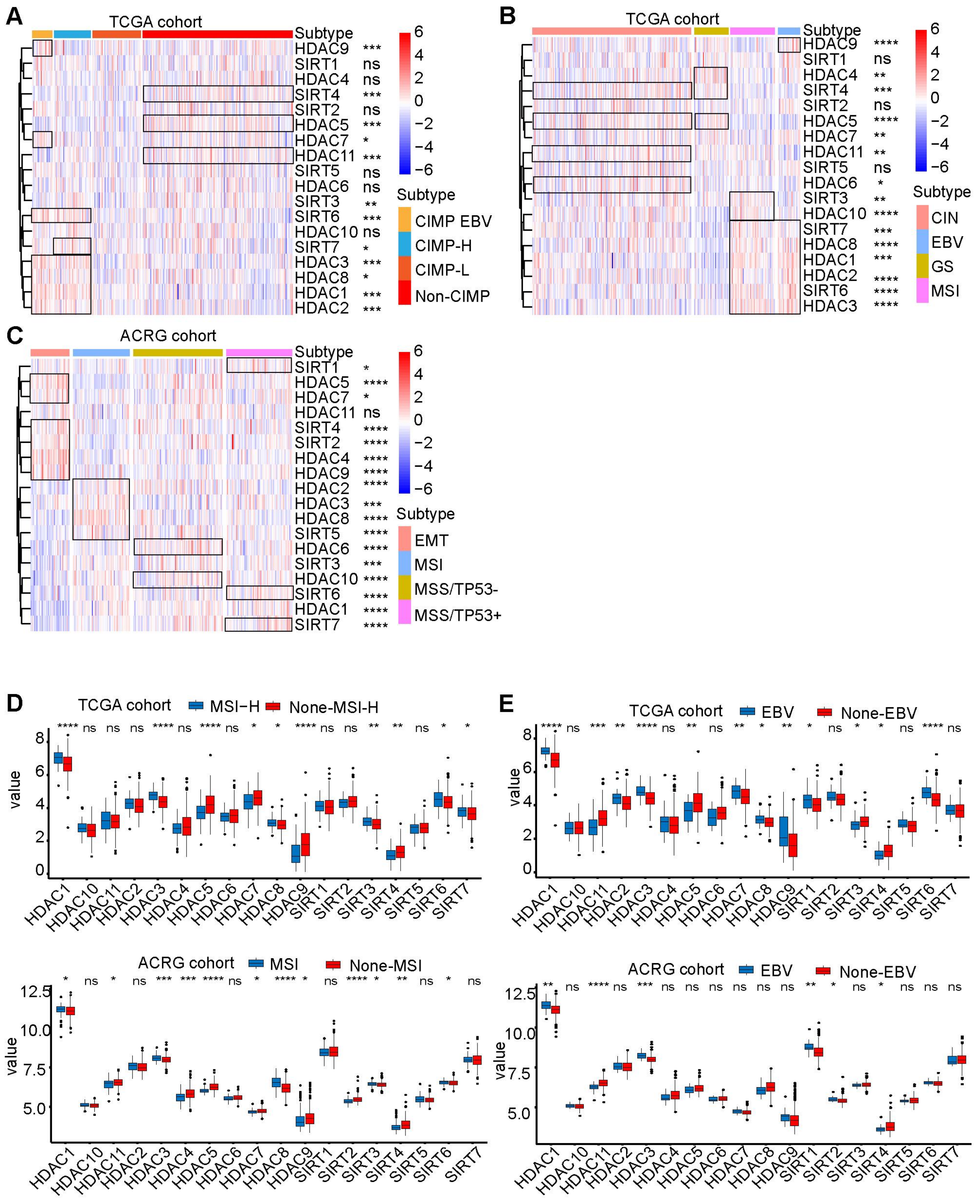

Figure S2 The relationship between the expression of HDACs and the subtypes of gastric cancer

(**A-C**) The differential expression of HDACs in gastric cancer subtypes. (**D**) The expression difference of HDACs in the MSI and MSS subtypes. (**E**) The expression difference of HDACs in EBV-positive and EBV-negative samples. * P<0.05, **P<0.01, ***P<0.001, ****P<0.0001.

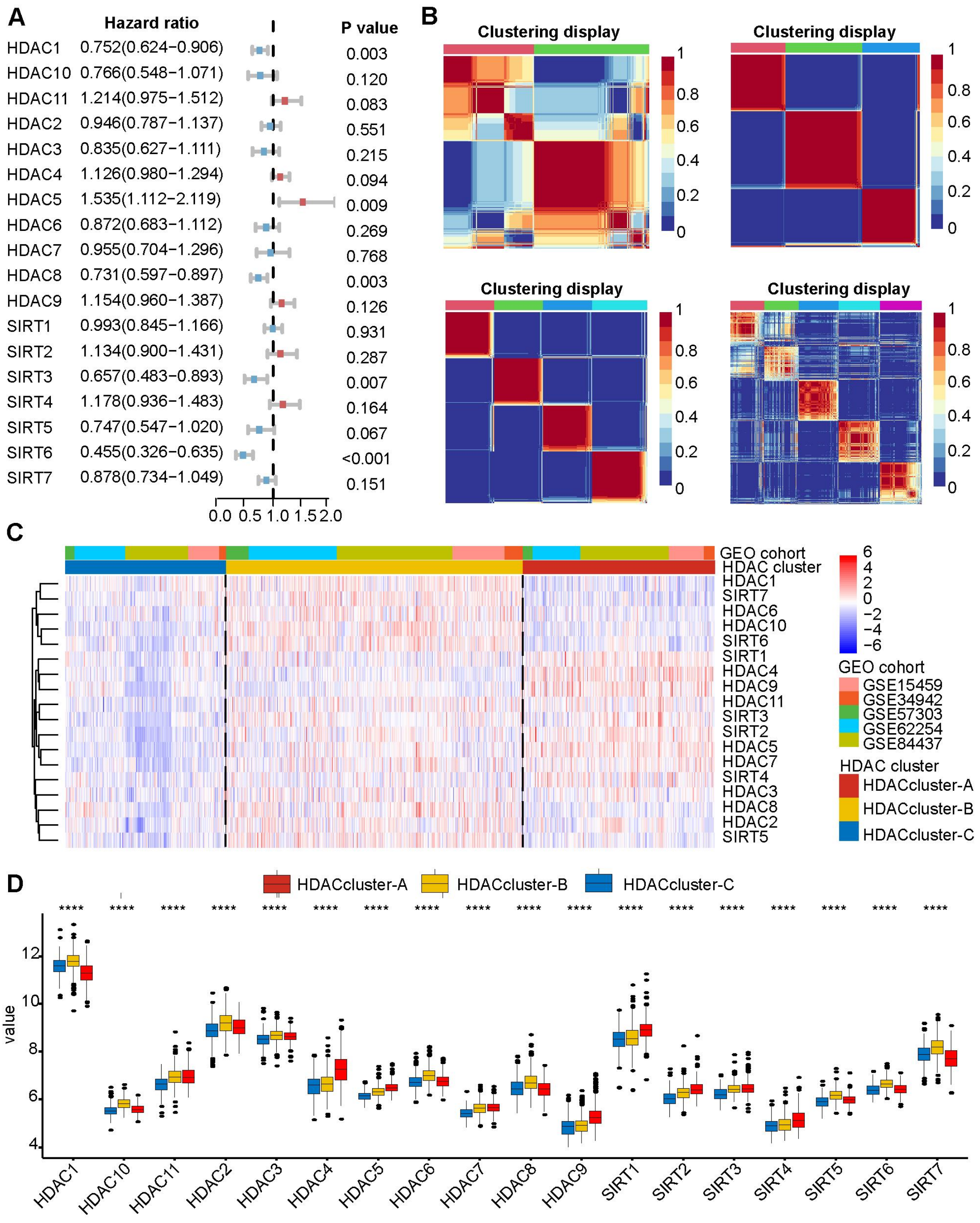

Figure S3 Landscape of HDACs in the GEO cohort

(A) Univariate Cox regression analysis to identify the relationship between HDACs and the prognosis of gastric cancer. (**B-C**) Based on the expression characteristics of HDACs, gastric cancer samples can be divided into three stable HDAC clusters by the NMF algorithm. (**D**) Expression of HDACs in different HDAC clusters. * P<0.05, **P<0.01, ***P<0.001, ****P<0.0001.

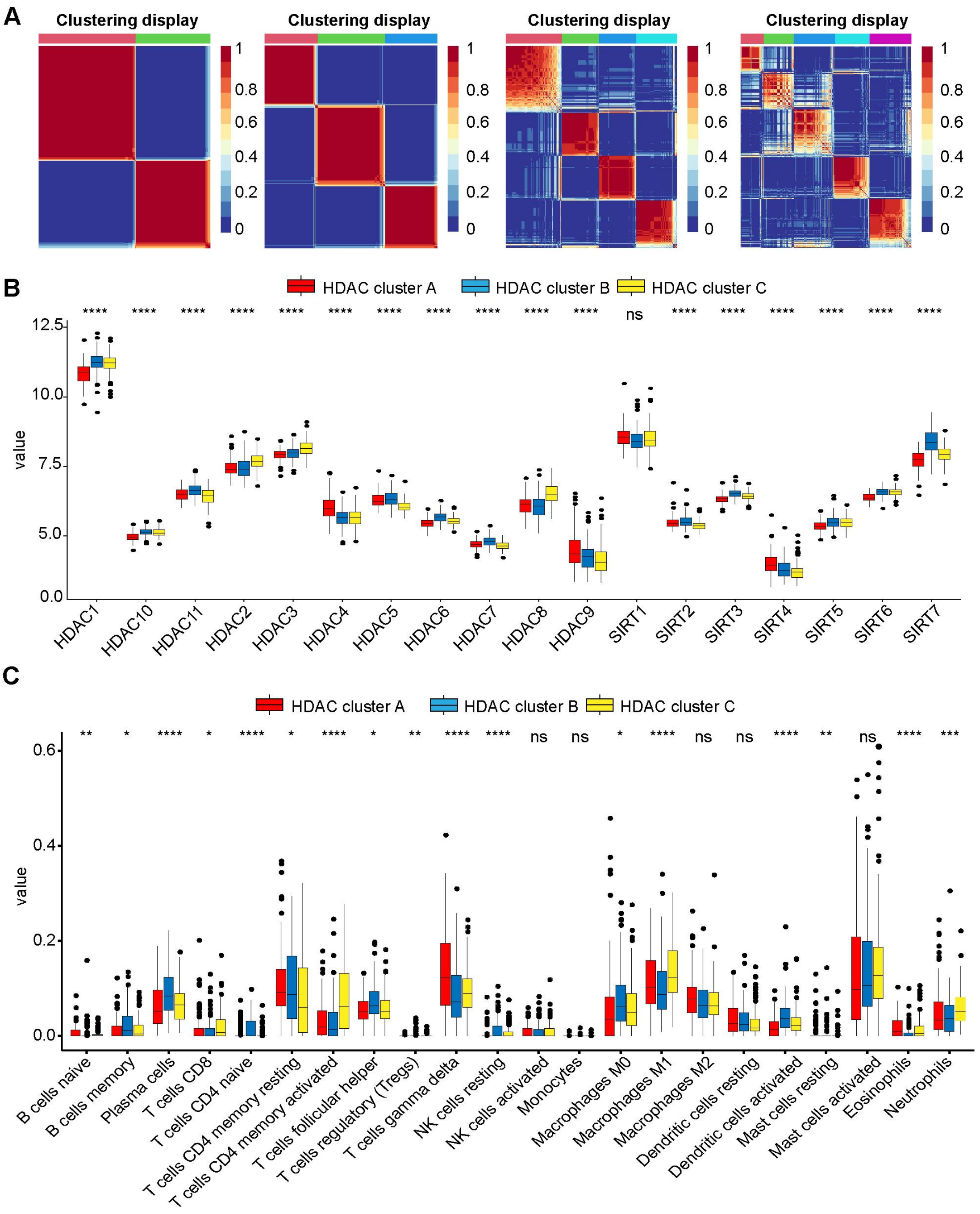

Figure S4 Gastric cancer samples can be divided into three subtypes based on the expression profile characteristics of HDACs (ACRG cohort)

(A) Identification of new subtypes of gastric cancer through the NMF algorithm. (**B**) Expression of HDACs in different HDAC clusters. (**C**) The difference in immune cell infiltration between different HDAC clusters. * P<0.05, **P<0.01, ***P<0.001, ****P<0.0001.

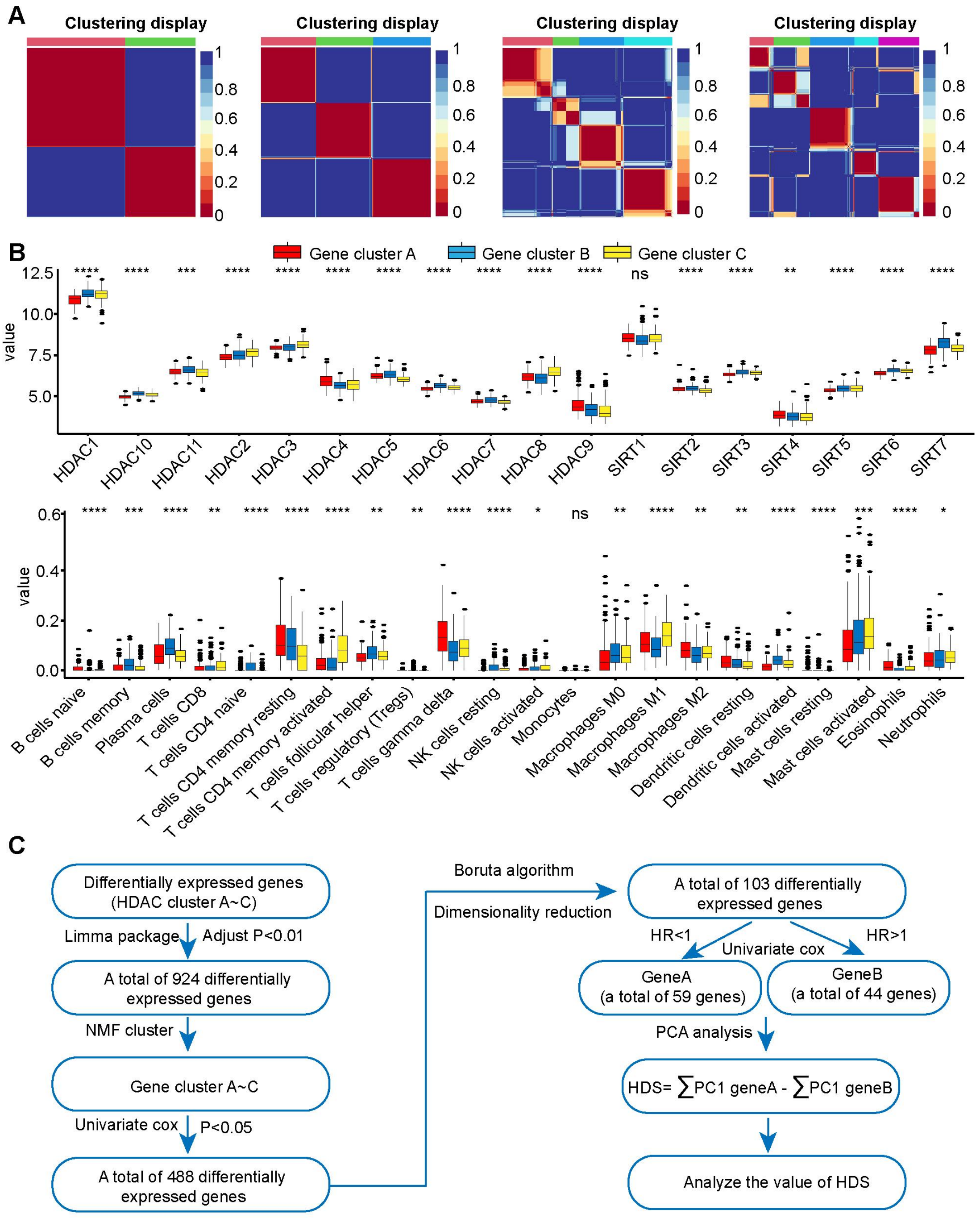

Figure S5 Construction of the HDS model

(A) Gastric cancer samples were divided into three gene clusters based on the genes that were significantly different between the HDAC clusters. (**B**) The differences in HDAC expression and immune cell infiltration levels in the three gene clusters. (**C**) Flow chart of HDS model construction. * P<0.05, **P<0.01, ***P<0.001, ****P<0.0001.

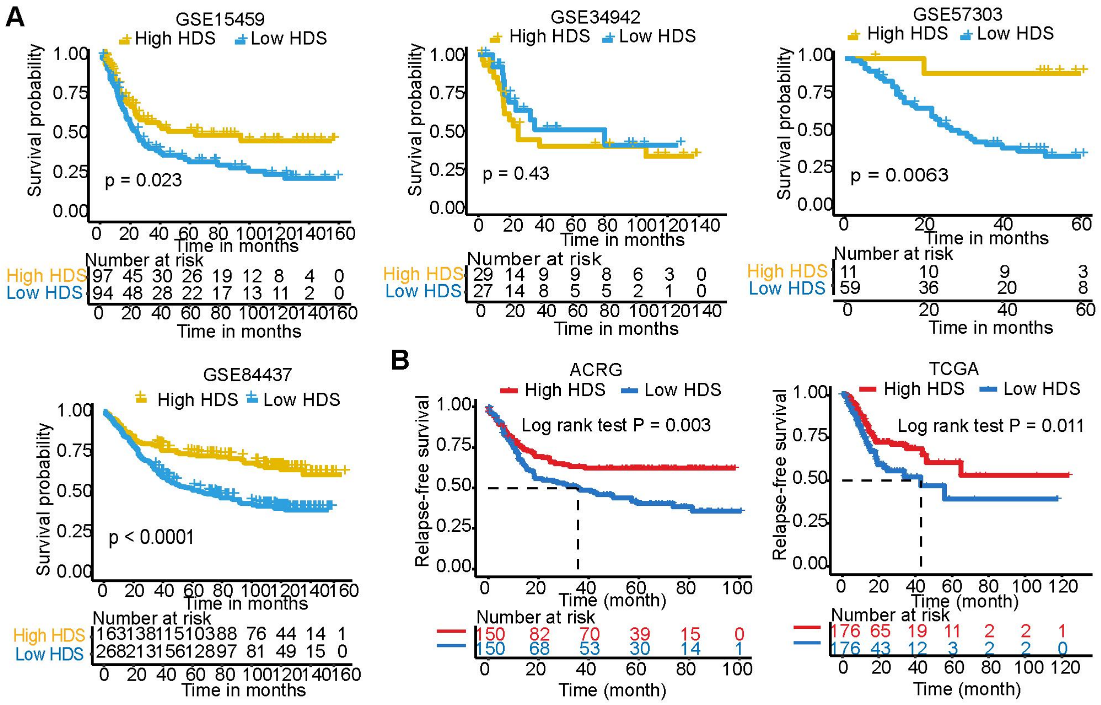

Figure S6 Prognostic value of the HDS in gastric cancer

(A) The overall survival difference between the high- and low-HDS groups. (B) The disease-free recurrence survival difference between the high- and low-HDS groups.

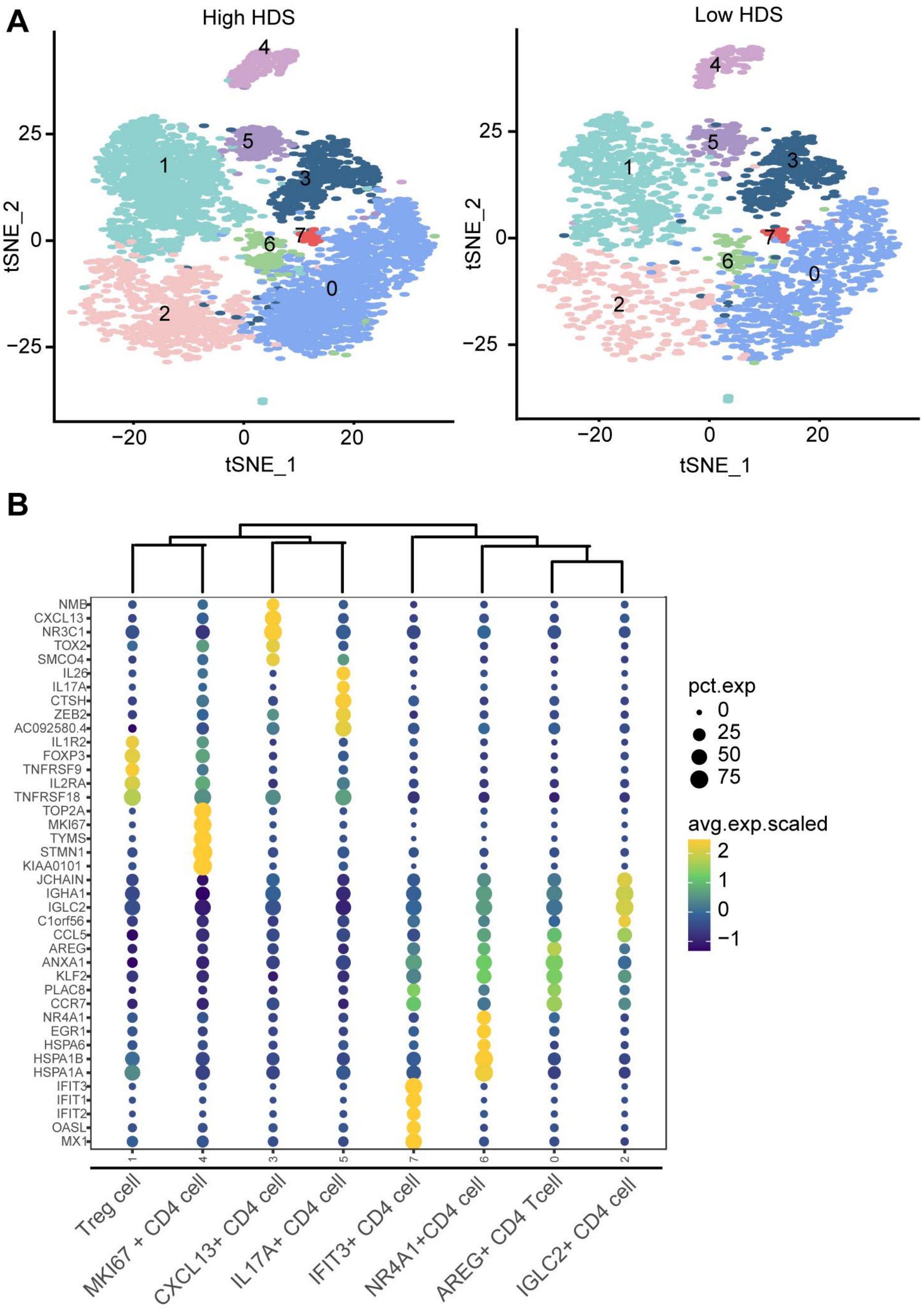

Figure S7 Reclustering and identification of CD4^{+} T cells

(A) The tSNE analysis revealed the distribution characteristics of CD4^{+} T-cell subsets in high- and low-HDS samples. (B) A bubble plot was used to show the top five marker genes for each subset.

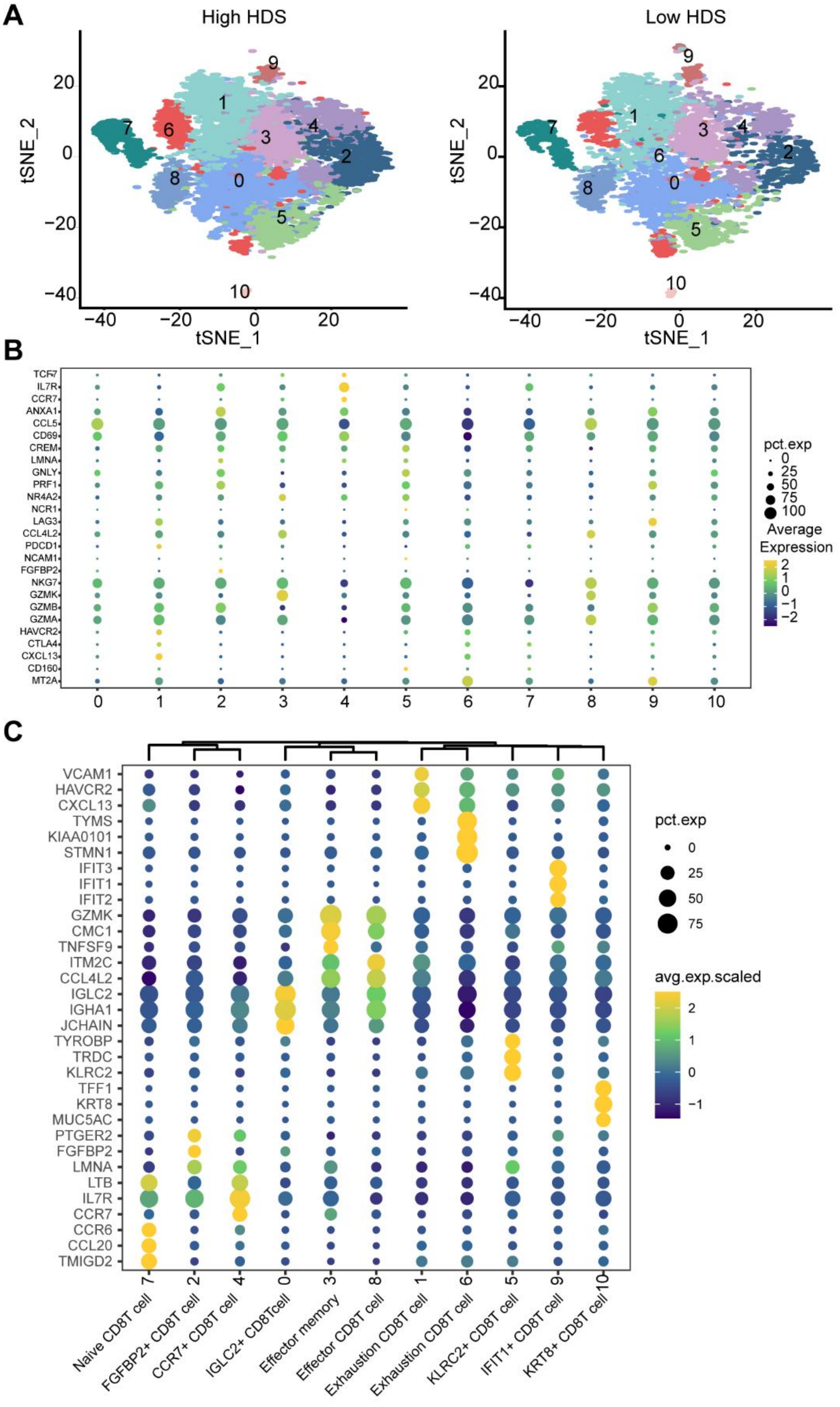

Figure S8 Reclustering and identification of CD8$^+$ T cells

(A)The tSNE analysis revealed the distribution characteristics of CD8$^+$ T-cell subsets in high- and low-HDS samples. (B) A bubble plot was used to show the expression of defined marker genes in each subset. (C) A bubble plot was used to show the top three marker genes for each subset.

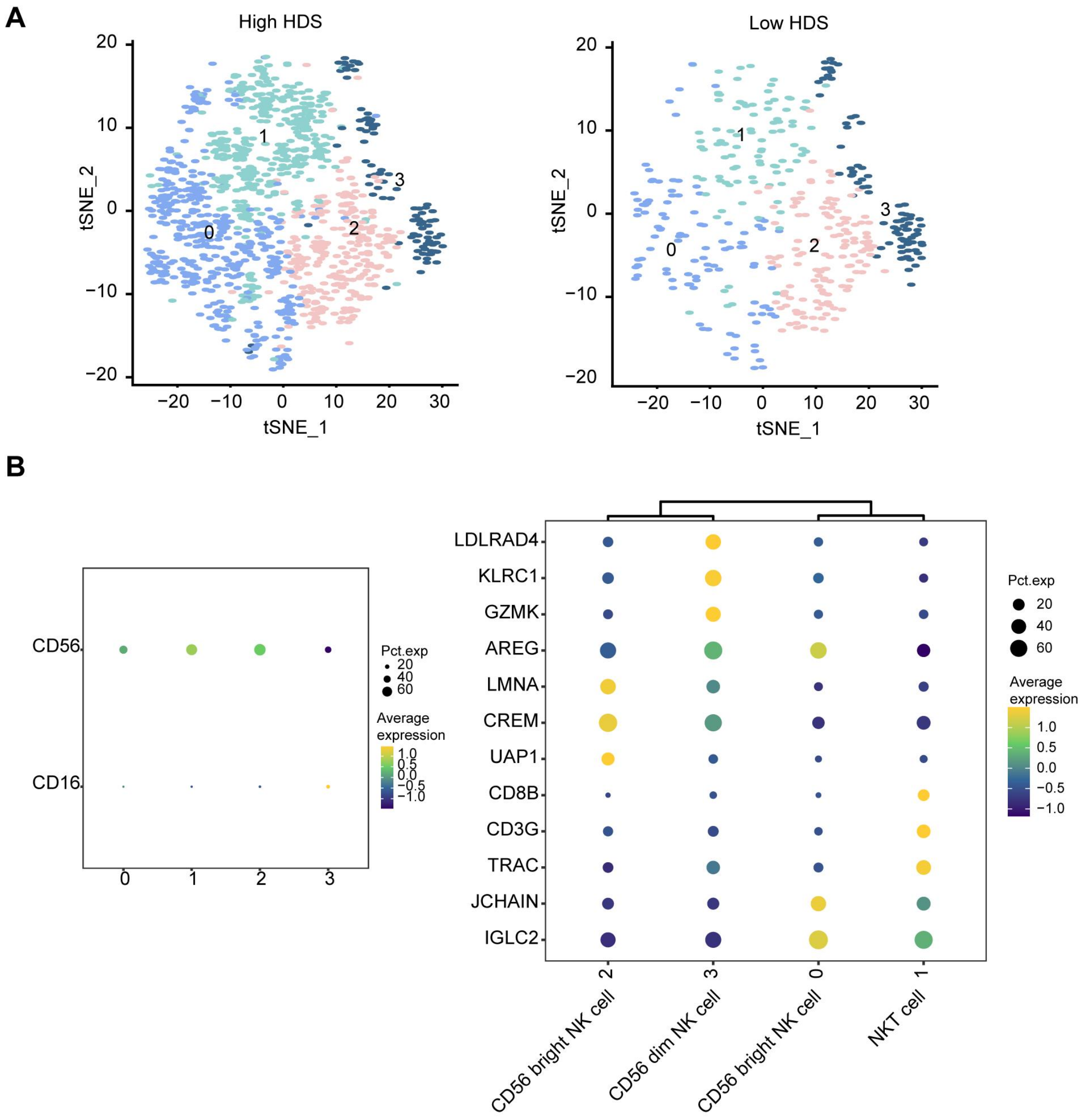

Figure S9 Reclustering and identification of NK cells

(A) The tSNE analysis revealed the distribution characteristics of NK-cell subsets in high- and low-HDS samples. (B) A bubble plot was used to show the expression of defined marker genes in each subset. (C) A bubble plot was used to show the top three marker genes for each subset.

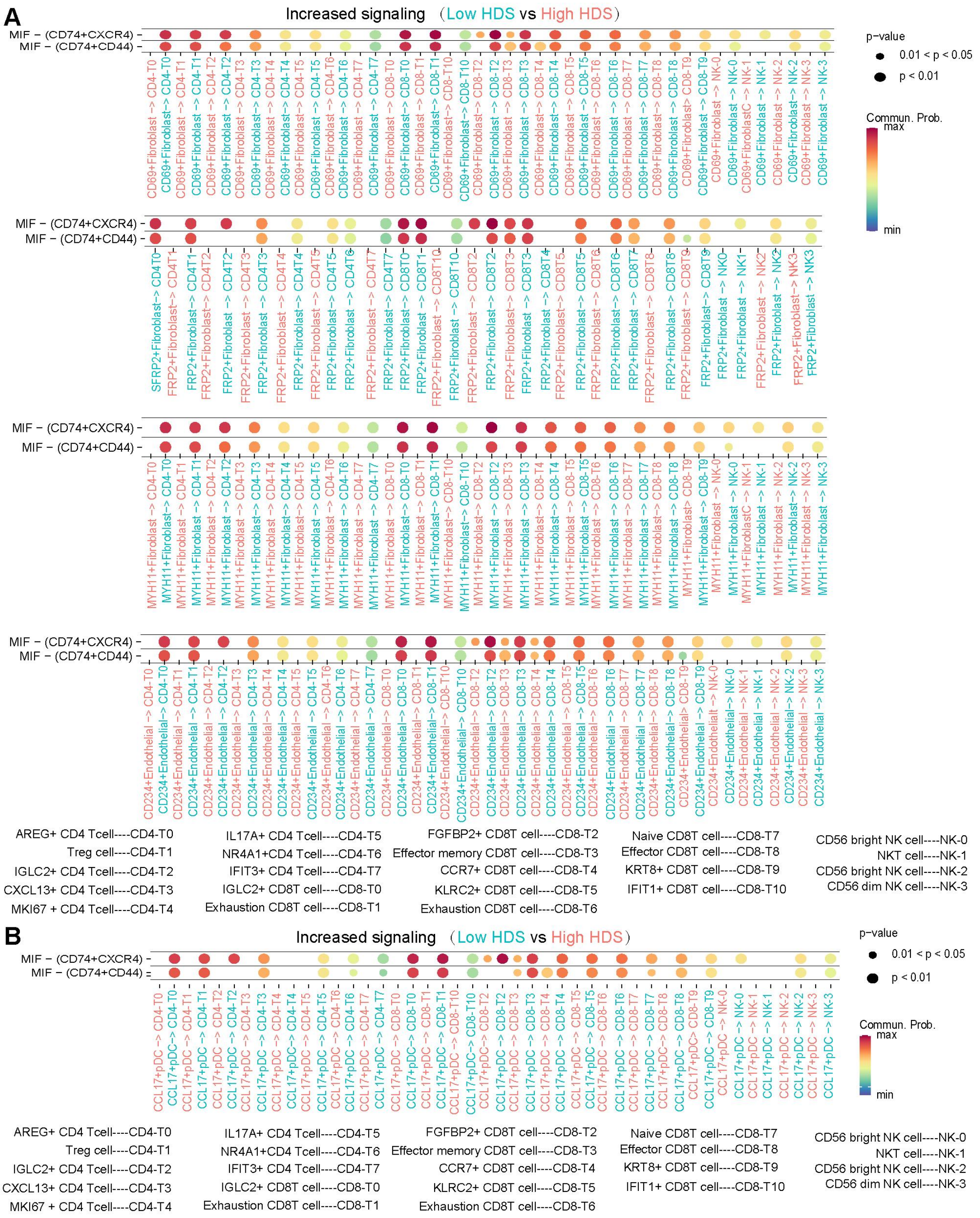

Figure S10 Differences in MIF signal communication between high- and low-HDS samples

(A) Difference analysis of communication between SFRP2^{+} fibroblasts, MYH11^{+} fibroblasts, CD234^{+} endothelial cells, CD69^{+} fibroblasts and T cells and NK cells in high- and low-HDS samples. (B) Difference analysis of communication between CCL17^{+} pDCs and T cells and NK cells in high- and low-HDS samples.

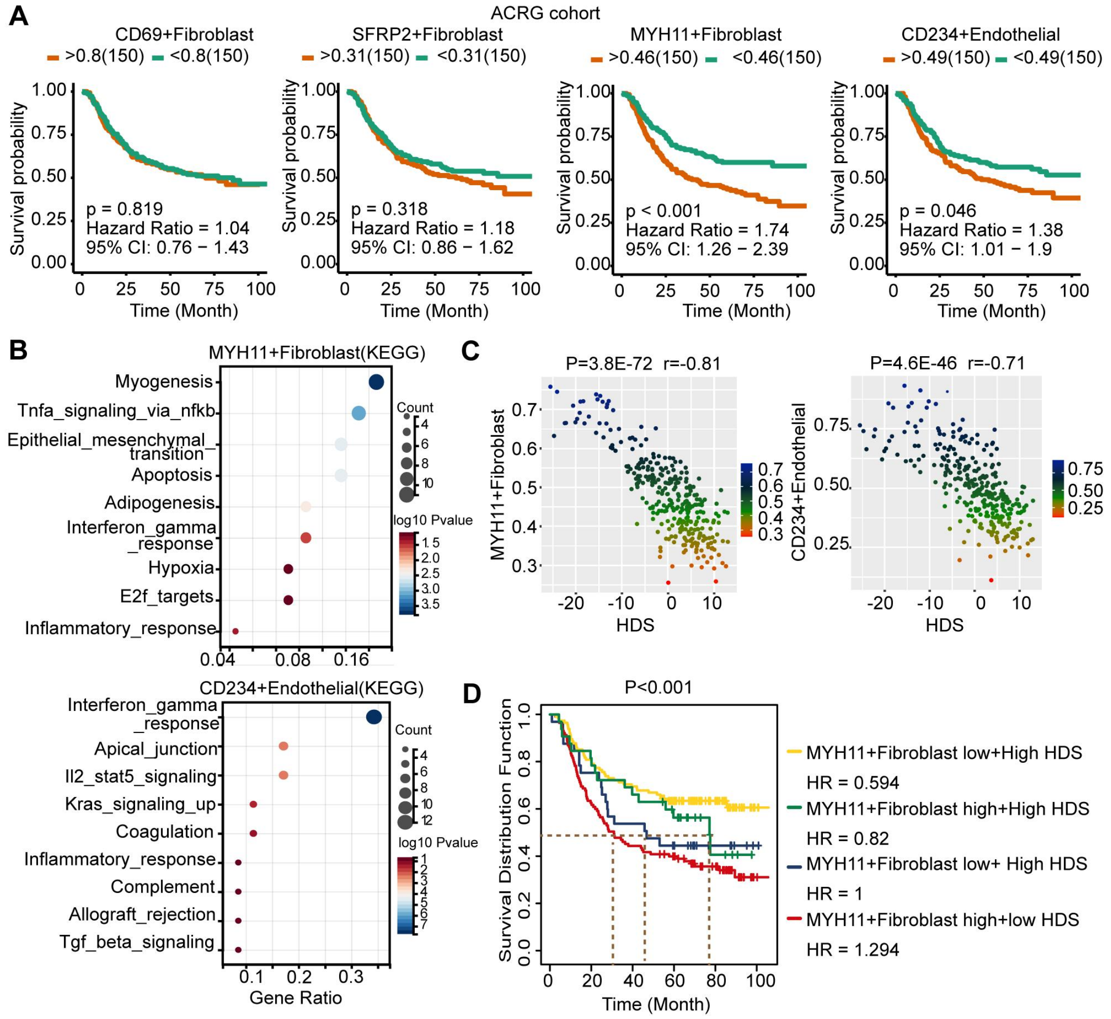

Figure S11 MYH11^{+} fibroblasts and CD234^{+} endothelial function and prognostic value

(A) Prognostic value of SFRP2^{+} fibroblasts, MYH11^{+} fibroblasts, CD234^{+} endothelial cells and CD69^{+} fibroblasts in the ACRG cohort. (B) MYH11^{+} fibroblasts and CD234^{+} endothelial function. (C) Correlation analysis of MYH11^{+} fibroblasts and CD234^{+} endothelial cells with the HDS. (D) Effect of MYH11^{+} fibroblasts and the HDS on the prognosis of gastric cancer.
